# How does light regulate plant regeneration?

**DOI:** 10.3389/fpls.2024.1474431

**Published:** 2025-01-29

**Authors:** Juan Han, Yapeng Li, Ye Zhao, Yuhan Sun, Yun Li, Zuodeng Peng

**Affiliations:** ^1^ State Key Laboratory of Tree Genetics and Breeding, Engineering Technology Research Center of Black Locust of National Forestry and Grassland Administration, College of Biological Sciences and Technology, Beijing Forestry University, Beijing, China; ^2^ The Key Laboratory for Silviculture and Conservation of Ministry of Education, College of Forestry, Beijing Forestry University, Beijing, China

**Keywords:** light, *de novo* shoot organogenesis, somatic embryogenesis, adventitious root regeneration, regulatory mechanism

## Abstract

Based on the totipotency and pluripotency of cells, plants are endowed with strong regenerative abilities. Light is a critical environmental factor influencing plant growth and development, playing an important role in plant regeneration. In this article, we provide a detailed summary of recent advances in understanding the effects of light on plant regeneration, with a focus on the fundamental processes and mechanisms involved in *de novo* shoot regeneration, somatic embryogenesis, and adventitious root formation. We focus on summarizing the effects of light intensity, light spectra, and photoperiod on these regeneration processes. Additionally, we propose the molecular mechanisms and regulatory networks underlying light-mediated plant regeneration. This article aims to deepen our understanding of the role of light in plant regeneration and to pave the way for future research on light-regulated regenerative processes in plants.

## 1 Introduction

Plants possess various mechanisms to adapt to their external environment, with regeneration being one of their most critical survival strategies. The totipotency of plant cells—a foundational principle in plant biology—was first discovered in 1958 when Steward and colleagues successfully regenerated entire plants from a single cell derived from the phloem tissue of *Daucus carota* L (Steward et al., 1958; Reinert, 1958). Plant regeneration is a process based on cellular totipotency, which enables plants to repair themselves and re-differentiate lost cells or form new organs near sites of injury. Plant regeneration is typically classified into three main processes: organogenesis, somatic embryogenesis, and tissue repair. First, in a medium supplemented with plant growth regulators, isolated plant tissues can undergo dedifferentiation to form callus, which then differentiates into complete plants. This process is referred to as *de novo* organogenesis. The ratio of auxin to cytokinin is a critical factor in determining the process of *de novo* organogenesis. When isolated plant tissues are in a higher ratio of cytokinin to auxin, adventitious shoots are regenerated near the wounds. Callus formation occurs under higher concentrations of auxin, while adventitious root (AR) is induced under lower concentrations of auxin (Skoog and Miller, 1957; Zhai and Xu, 2021). Different from plant organogenesis, somatic plant cells can also be induced to form somatic embryos under the influence of plant growth regulators or stress. These somatic embryos can further develop into complete plants. This process, known as somatic embryogenesis, is characterized by a high reproduction rate and good stability. As the mechanisms of plant regeneration have been increasingly elucidated, numerous plant regeneration systems have been established (Hua et al., 2013; Ikeda-Iwai et al., 2003; Gao et al., 2020; Tiidema and Truve, 2004). In addition, plants possess the ability to repair damaged tissues, restoring them to their original state, for example, the regeneration of new root tips following root tip excision and the healing of wounds during grafting (Feldman, 1976; Sena et al., 2009). Recent studies on plant regeneration, both mechanistic and applied, have been rapidly increasing. Emerging technologies have greatly advanced plant regeneration processes. With the continuous progress in plant genetic transformation and the development of technologies such as CRISPR-Cas9, it has become easier to conduct in-depth analyses of plant regeneration mechanisms and to apply plant regeneration technologies more widely (Lin et al., 2018; Wang et al., 2021; Zhang et al., 2024).

Light is a key environmental factor that influences plant growth and development, playing a role in processes such as seed germination, leaf development, circadian rhythms, and shade avoidance responses (Shah et al., 2021, 2024; Yan et al., 2024). Plants possess a variety of photoreceptors to detect light of different wavelengths. Phytochromes (PHYs: PHYA–PHYE) are sensitive to red and far-red light within the wavelength range of 600–760 nm. Cryptochromes (CRYs: CRY1, CRY2, and CRY-DASH), phototropins (PHOTs: PHOT1 and PHOT2), and zeitlupe (ZTL) primarily perceive ultraviolet light (320–400 nm) and blue light (400–500 nm) (Franklin and Quail, 2010; Christie, 2007; Pudasaini and Zoltowski, 2013; Kami et al., 2010). Photoreceptors transmit light signals to downstream regulatory factors, such as PHYTOCHROME-INTERACTING FACTORS (PIFs), ELONGATED HYOCOTYL 5 (HY5), and CONSTITUTIVE PHOTOMORPHOGENIC 1 (COP1) (Bhatnagar et al., 2020; Xu, 2020). These conserved light-responsive signaling factors then regulate downstream genes and proteins involved in plant regeneration. For example, HY5 inhibits the adventitious shoot regeneration by regulating the cytokinin-responsive factor *ARABIDOPSIS RESPONSE REGULATOR 12* (*ARR12*) and *WUSCHEL* (*WUS*), both of which are involved in the adventitious shoot regeneration of *Arabidopsis* (Dai et al., 2022). PHYB and PHYE promote somatic embryogenesis by regulating auxin synthesis genes, such as *AMIDASE 1* (*AMI1*), and jasmonic acid (JA)-responsive genes, such as *DE-ETIOLATED-2* (*DET2*) (Chan and Stasolla, 2023; Mira et al., 2023). Under dark conditions, PIFs directly bind to the promoters of *LATERAL ORGAN BOUNDARIES DOMAIN 16/29* (*LBD16/29*), which are involved in AR formation, thereby regulating the development of hypocotyl adventitious root (HAR) in *Arabidopsis* (Li et al., 2022b).

The effects of light on plant regeneration are broad. Light intensity, light spectra, and photoperiod are three attributes of light, all of which have complex and diverse effects on plant regeneration. Here, we discuss the effects of light on *de novo* shoot organogenesis, somatic embryogenesis, and AR regeneration. We summarize the molecular mechanisms and regulatory networks of light-regulated *de novo* shoot organogenesis, somatic embryogenesis, and AR regeneration, which provided important references for understanding and deeper investigation of light-influenced plant regeneration.

## 2 *De novo* shoot organogenesis

### 2.1 Basic process and molecular mechanisms of *de novo* shoot organogenesis

**Figure 1:**
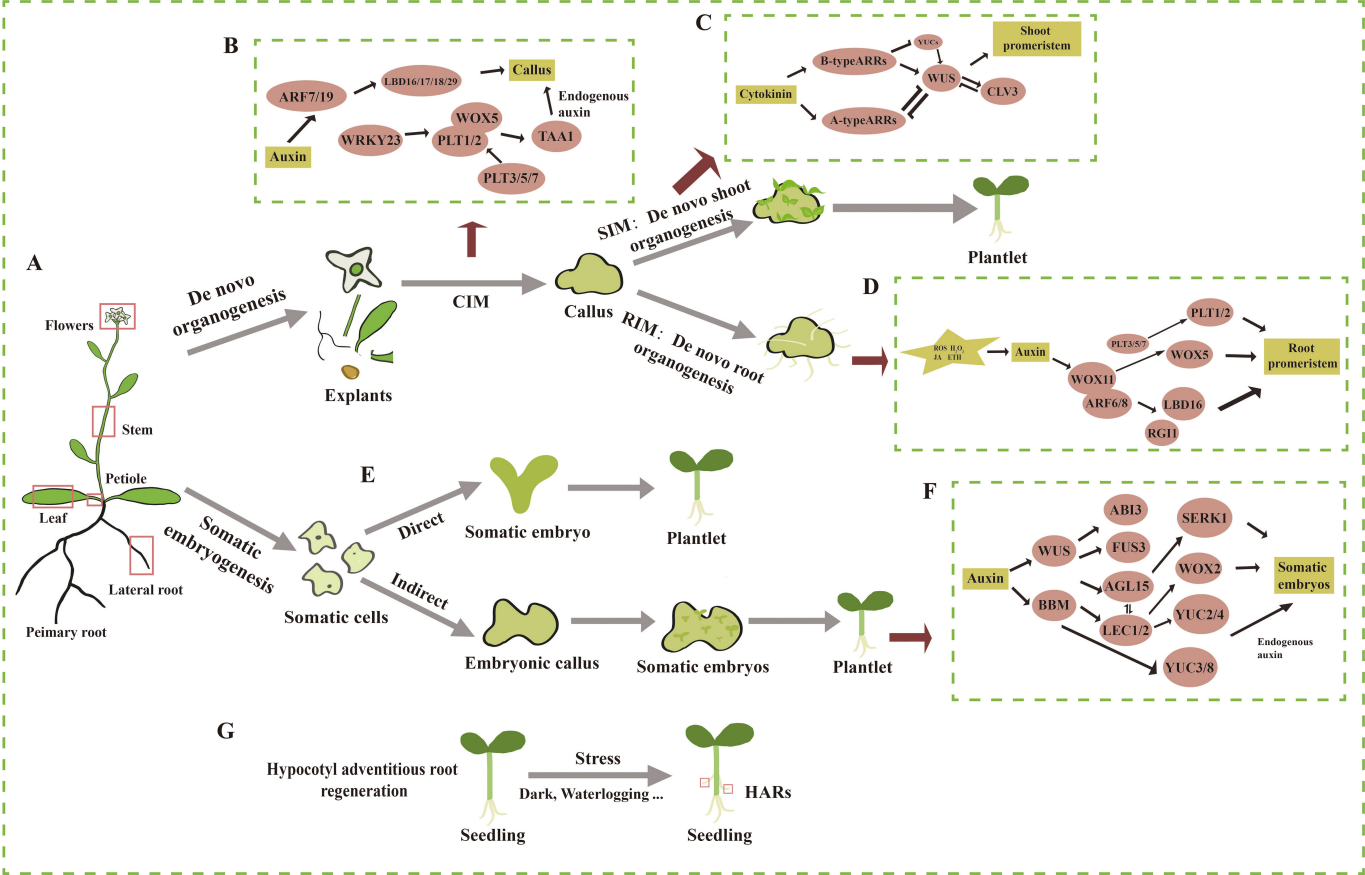
Overview of the processes and regulatory mechanisms of *de novo* shoot organogenesis, *de novo* root organogenesis, somatic embryogenesis, and hypocotyl adventitious root regeneration in the model plant *Arabidopsis thaliana*. **(A)** Schematic of an *Arabidopsis* plant where primary and lateral organs are shown. **(B)** The process and molecular mechanisms of dedifferentiation of explants *in vitro* on callus induction medium (CIM) to form pluripotent callus. **(C)** The process and molecular mechanisms of pluripotent callus regenerating adventitious shoots on shoot induction medium (SIM). **(D)** The process and molecular mechanisms of pluripotent callus regenerating adventitious roots on root induction medium (RIM). **(E)** The process of direct somatic embryogenesis. **(F)** The process and molecular mechanisms of indirect somatic embryogenesis. **(G)** The process of hypocotyl adventitious root (HAR) formation. The straight arrow represents activation, the connection of the blunt end represents suppression, and parallel lines indicate interactions.

Shoot organogenesis can also occur directly, bypassing the callus stage (Liu J. et al., 2022). In fact, even lateral root meristems can differentiate directly into shoot meristems without the formation of callus (Rosspopoff et al., 2017). This section focuses on the process of *de novo* shoot regeneration, which involves two key steps (**Figure 1**). First, isolated plant organs or tissues are placed on callus induction medium (CIM), which induces callus formation (**Figure 1B**). Callus typically originates from vascular cells or xylem pole pericycle cells. In response to auxin in the CIM, these cells undergo cell division, leading to the development of callus with characteristics of lateral root meristems (Atta et al., 2009; Sugimoto et al., 2010). At this stage, marker genes for root meristems, such as *AUXIN RESPONSE FACTOR 7/19* (*ARF7/19*), are expressed, which in turn induce the expression of four downstream transcription factors *LBD16/17/18/29* (Fan et al., 2012). These LBDs regulate callus formation through the modulation of cell wall modification, cell cycle, and cell division (Berckmans et al., 2011; Lee et al., 2013; Xu et al., 2018a, b). After callus formation, it must acquire pluripotency to proceed to the next stage of differentiation. PLETHORA (PLTs), a family of transcription factors involved in stem cell maintenance, play a key role in this process (Sang et al., 2018). WRKY23, located downstream of ARF7/19, indirectly activates the transcription of *WUSCHEL-RELATED HOMEOBOX 5* (*WOX5*) and *PLT1/2* by upregulating *PLT3* and *PLT7*, thereby promoting the acquisition of pluripotency in the callus. In *plt3/5/7* mutants, the expression of *PLT1* and *PLT2* is downregulated, resulting in the loss of pluripotency. Furthermore, the removal of *bHLH041*, induced by LBD16, alleviates its transcriptional repression of *PLT1*, *PLT2*, and *WOX5* (Xu et al., 2023; Kareem et al., 2015). Additionally, WOX5 and PLT1/2 interact to regulate the downstream expression of the auxin biosynthesis gene *TRYPTOPHAN AMINOTRANSFERASE OF ARABIDOPSIS 1* (*TAA1*), thereby promoting the synthesis of endogenous auxin in plants (Zhai and Xu, 2021).

Second, pluripotent callus undergoes continuous cell division and differentiation in the presence of cytokinin and auxin after being transferred to shoot induction medium (SIM) (**Figure 1C**) (Duclercq et al., 2011). Within 2–3 days of transfer to SIM, *WUS* is strongly expressed and plays a key role in the reconstitution of the early stem cell center, marking its formation (Zhang et al., 2017) (**Figure 1**). Cytokinin induces the expression of *WUS*, with ARR12 directly binding to the *WUS* promoter to enhance its expression (Zhang et al., 2017). In turn, WUS inhibits auxin signals and type A *ARR*s to facilitate *de novo* shoot organogenesis (Buechel et al., 2010; Negin et al., 2017). The expression of *WUS* is restricted to the base of the stem cell center, while *PIN-FORMED1* (*PIN1*) and *CUP-SHAPED COTYLEDON2* (*CUC2*) are expressed in the apical region, forming a protruding structure. WUS interacts with CLAVATA3 (CLV3) in a negative feedback loop, maintaining stem cell homeostasis while marking the zones of shoot initiation (Brand et al., 2002; Zhang et al., 2017). As *WUS* expression shifts upward, *CUC2* is expressed in the peripheral region of the stem cell center (Kareem et al., 2016), while *PIN1* is expressed in the outer cells of the meristem. At this point, a shoot apical meristem is established, which then differentiates into organs.

Epigenetic regulation has also been shown to play a role in regulating *de novo* shoot organogenesis. For instance, explants from the hypomethylated *fwa-1* mutant, which exhibits an elevated expression of *FLOWERING WAGENINGEN* (*FWA*), displayed reduced shoot regeneration compared to the wild type (Dai et al., 2021). Further experiments demonstrated that FWA inhibited adventitious shoot regeneration by binding to the promoter of *WOX9*. During shoot induction, histone deacetylase 19 (HDA19) suppressed the expression of *CUC2* by acetylating histones at the *CUC2* locus, thereby inhibiting adventitious shoot regeneration (Temman et al., 2023).

### 2.2 Effect of light on *de novo* shoot organogenesis

#### 2.2.1 Light intensity

Light intensity is a critical factor influencing *de novo* shoot organogenesis (**Table 1**). Plants can be categorized as either light-sensitive or light-demanding, depending on the light intensity required for successful regeneration (Rikiishi et al., 2015). It is generally believed that lower light intensities favor callus and adventitious shoot formation in light-sensitive plants. For example, Chen and co-workers found that low light intensity helps maintain the normal physiological state of callus induced from isolated leaves and stem segments of *Actinidia arguta*, preserving its green color and compact structure (Chen et al., 2019). However, as light intensity increased, the callus of *A. arguta* and *Haworthia* became browner, and the proliferation rate gradually decreased. Additionally, low light intensity was found to promote an increase in callus biomass (Sui et al., 2021). Lower light intensity also facilitates adventitious shoot formation in light-sensitive plants. Nameth and co-workers demonstrated that low-intensity light enhanced the regeneration of adventitious shoots from cotyledon explants of two *Arabidopsis* genotypes, ‘Ler’ and ‘DijG’. The efficiency of regeneration increased as light intensity decreased. This effect was attributed to the higher production of reactive oxygen species (ROS) and the depletion of the photoprotective pigment zeaxanthin at higher light intensities, leading to severe photo-oxidative damage (Nameth et al., 2013). A similar increase in adventitious shoot regeneration was observed in *Parthenium argentatum* when light intensity was reduced from 48 to 12 μmol·m^−2^·s^−1^, resulting in a twofold increase in both the number of explants producing shoots and the total number of shoots (Dong et al., 2006). The beneficial effect of low light intensity on adventitious shoot regeneration has also been observed in other species, including apple and *Phoenix dactylifera* L (Dobránszki and Teixeira Da Silva, 2011; Meziani et al., 2015).

**Table 1:** The effect of light on the *de novo* shoot organogenesis in plants.

The properties of light		Species	Function	Reference
Light intensity	0 lx/720 lx (0/13.3 μmol·m^−2^·s^−1^)	*Actinidia arguta*	Multiplication of leaf callus/stem callus	(Sui et al., 2021)
	10 μmol·m^−2^·s^−1^	*Haworthia*	Multiplication of callus	(Chen et al.,2019)
	3,000 lx (55.56 μmol·m^−2^·s^−1^)	*Nicotiana tabacum* L.	Induction rate of callus	(Siddique and Islam, 2018)
	60 μmol·m^−2^·s^−1^	*Allium hirtifolium*	Induction rate of shoot	(Farhadi et al., 2017)
	1,000 lx (18.52 μmol·m^−2^·s^−1^)	*Phoenix dactylifera* L.	Induction rate of shoot	(Meziani et al., 2015)
	20–25 μmol·m^−2^·s^−1^	*Arabidopsis thaliana*	Induction rate of shoot	(Nameth et al., 2013)
	150 μmol·m^−2^·s^−1^	*Linum usitatissimum* L.	Induction rate of shoot	(Caillot et al., 2009)
	12 μmol·m^−2^·s^−1^	*Parthenium argentatum*	Induction rate of shoot	(Dong et al., 2006)
	24 μmol·m^−2^·s^−1^	*Cistanche deserticola*	Induction rate of callus	(Ouyang et al., 2003)
	50 μmol·m^−2^·s^−1^	*Cucumis melo* L.	Induction rate of shoot	(Leshem et al., 1995)
	3,000 lx (55.56 μmol·m^−2^·s^−1^)	*Brassica oleracea* var. *botrytis* L.	Induction rate of shoot	(Kumar et al., 1993)
Light spectra	Blue	*Arachis hypogaea*	Promotes adventitious shoot regeneration	(Assou et al., 2023)
	Blue and red	*Salvia bulleyana*	Promote adventitious shoot regeneration	(Krzemińska et al., 2023)
	Blue	*Operculina turpethum* L.	Promote multiplication of callus	(Biswal et al., 2022)
	Blue and red	*Hyoscyamus reticulatus*	Promote multiplication of callus	(Hassanpour, 2022)
	Blue and red	*Rubus fruticosus* L. *Rubus idaeus* L.	Promote adventitious shoot regeneration	(Loshyna et al., 2022)
	Blue or red	*R. fruticosus* L. *R. idaeus* L.	Inhibit adventitious shoot regeneration	(Loshyna et al., 2022)
	Red/far-red	*A. thaliana*	Promote adventitious shoot regeneration	(Dai et al., 2022)
	Blue/white	*Cnidium officinale* Makino	Promote embryogenic callus regeneration	(Adil et al., 2019)
	Red/red and blue	*C. officinale* Makino	Promote non-embryogenic callus regeneration	(Adil et al., 2019)
	Red	*Rhodiola imbricata*	Promote multiplication of callus	(Kapoor et al., 2018)
	White	*Ajuga multiflora*	Promote adventitious shoot regeneration	(Jeong and Sivanesan, 2018)
	Red and blue	*Swertia chirata*	Promote adventitious shoot regeneration	(Dutta Gupta and Karmakar, 2017)
	Red or green	*Populus alba × Populus berolinensis*	Inhibit adventitious shoot regeneration	(Wang et al., 2008)
	White or yellow	*P. alba × P. berolinensis*	Promote adventitious shoot regeneration	(Wang et al., 2008)
	Dark or red or far-red	*Hordeum vulgare* L. ‘K3’	Promote adventitious shoot regeneration	(Rikiishi et al., 2008)
	White	*Petunia hybrida*	Promote adventitious shoot regeneration	(Reuveni and Evenor, 2007)
	Red or blue or dark	*P. hybrid*	Inhibit adventitious shoot regeneration	(Reuveni and Evenor, 2007)
	Red or far-red	*Solanum lycopersicum* L.	Promote adventitious shoot regeneration	(Lercari and Bertram, 2004)
	Blue	*C. deserticola*	Promote multiplication of callus	(Ouyang et al., 2003)
	Red or white	*Begonia × erythrophylla*	Promote adventitious shoot regeneration	(Burritt and Leung, 2003)
	Blue or far-red or dark	*Begonia × erythrophylla*	Inhibit adventitious shoot regeneration	(Burritt and Leung, 2003)
	Blue	*Eutrema salsugineum*	Inhibit multiplication of callus	(Pashkovskiy et al., 2018)
Photoperiod	16/8 h (light/dark)	*S. lycopersicum* L.	Promote adventitious shoot regeneration	(Song et al., 2023)
	Initial low-fluence red light or darkness	*A. thaliana*	Promote adventitious shoot regeneration	(Wei et al., 2020)
	Early 2–24-h darkness	*A. thaliana*	Promote adventitious shoot regeneration	(Nameth et al., 2013)
	Darkness for 20 d	*Citrus reticulata* Blanco	Promote adventitious shoot regeneration	(Zeng et al., 2009)
	Darkness for 7 d	*H. vulgare* L. ‘K3’, ‘K5’	Promote adventitious shoot regeneration	(Rikiishi et al., 2008)
	Darkness for 7 d	*H. vulgare* L. ‘LN’	Promote adventitious shoot regeneration	(Rikiishi et al., 2008)
	Darkness for 5 weeks	*Prunus serotina*	Promote multiplication of callus	(Espinosa et al., 2006)
	Darkness for 3 weeks	*Prunus persica* L.	Promote adventitious shoot regeneration	(Gentile et al., 2002)
	Darkness for 20 d	*Malus domestica* Borkh	Promote adventitious shoot regeneration	(Caboni et al., 2000)
	Darkness for 15 d	*Erigeron breviscapus*	Promote adventitious shoot regeneration	(Xing et al., 2008)
	16/8 h (light/dark)	*Oryza sativa* L.	Promote adventitious shoot regeneration	(Liu et al., 2001)
	16/8 h (light/dark)	*P. hybrida*	Promote adventitious shoot regeneration	(Reuveni and Evenor, 2007)

In contrast to light-sensitive plants, light-promoted plants require high-intensity light for callus and adventitious shoot formation. For example, the rate of callus induction in *Nicotiana tabacum* L. was higher under a high light intensity of 3,000 lux (approximately 55.56 μmol·m^−2^·s^−1^) compared to dark conditions. Callus developed in the dark appeared watery and glossy silver in color, with fewer embryogenic potential (Siddique and Islam, 2018). Similarly, more calli were induced from hypocotyls of *Linum usitatissimum* L. at 150 μmol·m^−2^·s^−1^ compared to 75 μmol·m^−2^·s^−1^ (Caillot et al., 2009). This increase in callus formation was attributed to the higher sucrose utilization by the explants under high light intensity (Farhadi et al., 2017). Additionally, explants of *Cucumis melo* L. and *Brassica oleracea* var. *botrytis* L. produced a greater number of adventitious shoots under high light intensity (Kumar et al., 1993; Leshem et al., 1995). In summary, light intensity influences the dedifferentiation process of explants by affecting the state and browning degree of the callus, and it also impacts the differentiation process of adventitious shoots by modulating sucrose utilization in the explants.

#### 2.2.2 Light spectra

Blue, red, far-red, and mixed light wavelengths are extensively utilized in plant regeneration studies (**Table 1**). The explants of plant species complete their regeneration process by responding to different photoreceptors under various light spectra. Studies have shown that blue, red, or a combination of red and blue light can significantly promote the regeneration of adventitious shoots. For example, callus of *Cnidium officinale* Makino grown under blue light exhibited a compact texture and showed shoot regeneration after sub-culturing, while friable and watery non-regenerative callus was observed under dark or red light (Adil et al., 2019). Blue light has also been shown to enhance the antioxidant activity in the callus of *Operculina turpethum* L. and *Eutrema salsugineum*. In *O. turpethum* L., the levels of total phenols and flavonoids increased, and in *E. salsugineum*, the activities of key antioxidant enzymes, such as catalase (CAT) and peroxidase (POD), were higher in callus grown under blue light compared to that cultivated under white light (Biswal et al., 2022; Pashkovskiy et al., 2018). Additionally, blue light promoted biomass accumulation in the callus of *O. turpethum* L. and *Cistanche deserticola* (Ouyang et al., 2003). A similar effect was observed in the regeneration of *Arachis hypogaea*, where leaf explants formed only callus that could not differentiate under white light but formed adventitious shoots under blue light (Assou et al., 2023). Red light promoted callus biomass accumulation in *Rhodiola imbricata* and *Hordeum vulgare* L (Kapoor et al., 2018; Rikiishi et al., 2008). Active phytochromes under red light stimulated the synthesis and activity of growth-related enzymes, which also promoted the formation of shoot meristems in the callus of *Begonia × erythrophylla*. Each explant produced more than 25 shoots (Burritt and Leung, 2003). Hypocotyl explants of *Solanum lycopersicum* L. exhibited higher regeneration efficiency under red light, with adventitious shoot regeneration rates significantly lower in the *phyb* mutant compared to white light conditions (Lercari and Bertram, 2004). Shoot tips of *Swertia chirata* showed the highest chlorophyll, carotenoid, and polyphenol contents as well as the greatest efficiency of adventitious shoot regeneration under mixed red and blue light (Dutta Gupta and Karmakar, 2017). Mixed red and blue light also increased the adventitious shoot regeneration rate in *Rubus fruticosus* L. and *Rubus idaeus* L. by promoting cell division, maintaining the redox state (Hassanpour, 2022), and regulating the cell cycle (Kwon et al., 2015; Loshyna et al., 2022). Additionally, white or yellow light facilitated adventitious shoot regeneration in *Populus alba* × *Populus berolinensis*, whereas green light inhibited this process of *P. alba* × *P. berolinensis* (Wang et al., 2008).

In conclusion, light spectra significantly influence callus growth, proliferation, and antioxidant activity by modulating the activity of photosensitive pigments, which in turn upregulate genes encoding growth-related enzymes. The application of appropriate light spectra enhances cell viability and regulates the cell cycle, thereby ensuring that the callus remains capable of both proliferation and differentiation. Moreover, light spectra play a crucial role in the regeneration of adventitious shoots by affecting photosynthesis and promoting the formation of shoot meristems. Understanding the molecular mechanisms through which light spectra regulate these processes is essential for comprehending the role of light in plant regeneration.

#### 2.2.3 Photoperiod

The 16/8-h light/dark photoperiod is crucial for plant growth and development, and different photoperiods have distinct effects on adventitious shoot regeneration (**Table 1**). In some plant species, *de novo* shoot organogenesis is promoted in darkness. For example, in *Arabidopsis*, darkness treatment led to the regeneration of more adventitious shoots from excised explants compared to the 16/8-h photoperiod (Nameth et al., 2013). The inhibitory effect of light at culture initiation on the adventitious shoot regeneration was alleviated by the addition of *N*-1-naphthylphthalamic acid (NPA), an auxin polar transport inhibitor. Ethylene synthesis was also regulated by light, with ethylene levels increasing under darkness. The addition of the ethylene precursor, 1-aminocyclopropane-1-carboxylic acid (ACC), further promoted adventitious shoot formation under darkness (Nameth et al., 2013). The above suggests that light photoperiod regulates adventitious shoot regeneration by influencing auxin polar transport and ethylene levels. Wei and co-workers demonstrated that genes involved in the synthesis and signaling of auxin, cytokinin, and ethylene were differentially expressed in darkness during the culture initiation. Additionally, key factors directly involved in adventitious shoot regeneration, such as *LBD16*, *PLT3*, *WOX5*, *WUS*, and *SHOOT MERISTEMLESS* (*STM*), were highly expressed under darkness in *Arabidopsis* (Wei et al., 2020). In *Erigeron breviscapus*, darkness for 15 days resulted in a significant increase in adventitious shoots number, with a regeneration efficiency of 82.6% (Xing et al., 2008). Woody plants, which typically have longer cultivation periods, are prone to producing phenolic compounds and oxidative enzymes during regeneration. However, after 20 days of darkness, the shoot regeneration rate in *Citrus reticulata* Blanco reached 100%, with an average of 13.2 shoots regenerated per explant. Under a 16/8-h photoperiod, the regeneration rate was only 72.5%, with an average of 7.8 shoots regenerated per explant (Zeng et al., 2009). Similarly, darkness also promoted the regeneration of adventitious shoots in *Malus × domestica* Borkh and *Prunus persica* L (Caboni et al., 2000; Gentile et al., 2002).

Photoperiods with extended light durations have also been shown to promote adventitious shoot regeneration in some plant species. In the *S. lycopersicum* cultivar Micro-Tom, no significant difference in regeneration was observed between tomato leaf explants pre-cultured under darkness for 8 days and those under 16/8-h photoperiod (Song et al., 2023). However, under a 16/8-h photoperiod, numerous light-regulated chlorenchyma cells containing chloroplast-like structures appeared near the sites of adventitious shoot primordium formation. When the photosynthesis inhibitor 3-(3,4-dichlorophenyl)-1,1-dimethylurea (DCMU) was applied to inhibit photosynthesis in these cells, the number of adventitious shoots regenerated from the leaves decreased. This indicates that these cells provide essential energy for the formation of adventitious shoot primordia and highlights the role of photosynthesis in adventitious shoot formation. Additionally, the highest expression levels of regeneration-related genes, such as *PLT3* and *STM*, are observed under a 16/8-h photoperiod (Song et al., 2023). In *Oryza sativa* L., during the regeneration of adventitious shoots from pluripotent callus, the number of adventitious shoots increased progressively with longer light durations and reached its maximum under a 16/8-h photoperiod (Liu et al., 2001). In summary, photoperiod affects plant regeneration by influencing the synthesis and transport of various hormones, photosynthesis, the synthesis of phenolic compounds, and cell fate transition during adventitious shoot regeneration.

#### 2.2.4 Molecular mechanisms of light-regulated *de novo* shoot organogenesis

**Figure 2:**
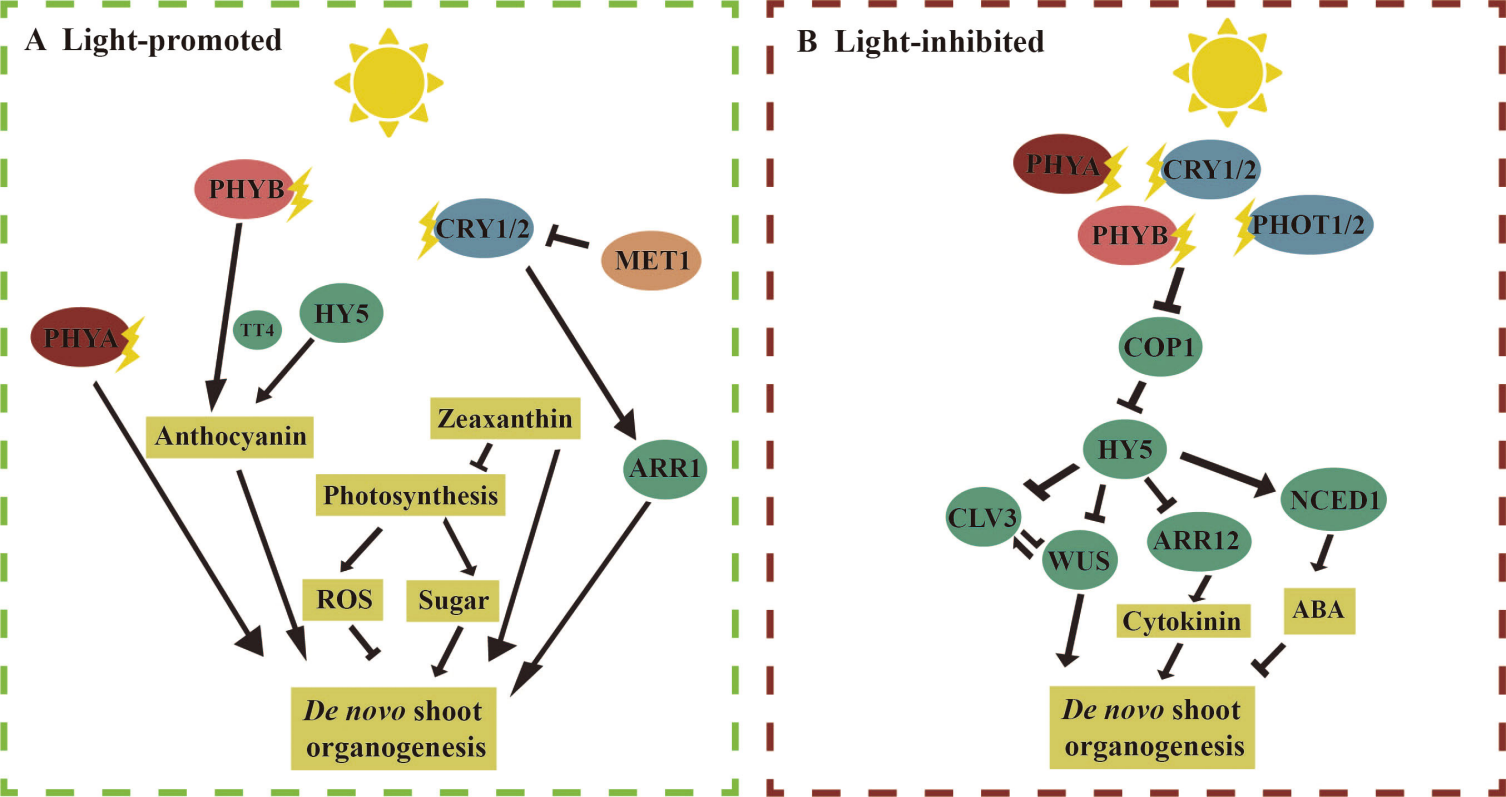
Molecular mechanism of light regulation of *de novo* shoot organogenesis. [**(A)**, left] Light promotes *de novo* shoot organogenesis: in *Arabidopsis*, under light conditions, compared to the wild type, the phytochrome A (*phyA*) mutant produced fewer adventitious shoots from cotyledon explants. Phytochrome B (PHYB) and ELONGATED HYPOCOTYL 5 (HY5) receive light signals and promote anthocyanin synthesis by regulating the expression of the anthocyanin synthase gene *TRANSPARENT TESTA 4* (*TT4*), which in turn promotes *de novo* shoot regeneration. CRY1/2 enhances the *Arabidopsis response factor 1* (*ARR1*) to promote adventitious shoot regeneration. Additionally, in both tomato and *Arabidopsis*, light may promote the synthesis of zeaxanthin, reactive oxygen species (ROS), and sugar to support adventitious shoot regeneration. [**(B)**, right] Light inhibits *de novo* shoot organogenesis: PHYA/B and CRY1/2 regulate light signaling under blue and red light by inhibiting CONSTITUTIVE PHOTOMORPHOGENIC 1 (COP1) activity and stabilizing HY5. During adventitious shoot regeneration in *Arabidopsis* root explants, *HY5* can directly bind to the promoters of WUSCHEL (*WUS*) and *CLAVATA3* (*CLV3*) to suppress their expression. *HY5* can also inhibit the expression of *Arabidopsis response factor 12* (*ARR12*) by binding to its promoter, further suppressing *WUS* expression. In barley, it is found that abscisic acid (ABA) inhibits adventitious shoot regeneration, and under light conditions, the expression level of the gene *9-cis-epoxycarotenoid dioxygenase 1* (*NCED1*), which encodes ABA synthase, increases. The straight arrow represents activation, the connection of the blunt end represents suppression, and parallel lines indicate interactions.

Light regulates *de novo* shoot organogenesis through a complex network that involves both positive and negative regulatory pathways. Multiple regulatory pathways can exist for the same light-responsive factors, acting through both positive and negative mechanisms. Here, we first review the molecular mechanisms associated with light-promoted adventitious shoot regeneration (**Figure 2A**). Phytochromes PHYA, PHYB, and CRY1 are the primary receptors that sense light signals and directly regulate downstream factors involved in adventitious shoot regeneration. In *Arabidopsis* ‘Ler’ and tomato, the ability to regenerate adventitious shoots was significantly impaired in the *phyA* mutant (Lercari and Bertram, 2004; Nameth et al., 2013; Saitou et al., 1999). Both PHYB and HY5 directly regulated the anthocyanin synthase gene *TT4*, promoting adventitious shoot regeneration. It was found that the regeneration rate of adventitious shoots was significantly lower in *hy5* and *tt4* mutants compared to the wild type, and anthocyanins were absent in *phyB* mutants (Nameth et al., 2013). The *cry1* mutant in *Arabidopsis* showed a reduced ability to regenerate adventitious shoots compared to the wild type. CRY1 in *Arabidopsis* promoted adventitious shoot regeneration by enhancing the expression of the cytokinin response factor *ARR1*. In the *cry1* mutant, both adventitious shoot regeneration and the expression of *ARR1* were significantly reduced (Shim et al., 2021). Additionally, immature embryos of the bare cultivar ‘LN’ exhibited higher auxin content under a 16/8-h photoperiod, suggesting that light may influence adventitious shoot regeneration by regulating auxin levels (Rikiishi et al., 2015). Light also indirectly affected adventitious shoot regeneration in *Arabidopsis* and tomato by modulating photosynthesis, ROS, and photoprotective zeaxanthin. For example, the photosynthesis inhibitor DCMU significantly reduced the rate of callus regeneration. Furthermore, reduced levels of photoprotective zeaxanthin were observed in *non-photochemical quenching 1* (*npq1*) mutants, which caused a significant reduction in adventitious shoot regeneration from cotyledons both in light and darkness (Nameth et al., 2013; Song et al., 2023). These pathways and the key factors involved in these processes remain to be further explored. Additionally, epigenetic regulation plays a role in light-induced adventitious shoot regeneration. For example, the DNA methyltransferase MET1 inhibited the expression of the *CRY1* by methylating the DNA at the *CRY1* locus, thereby reducing adventitious shoot regeneration in *Arabidopsis*. In contrast, the *met1* mutant displayed enhanced adventitious shoot regeneration (Shim et al., 2021).

Next, we discuss the mechanisms involved in the inhibition of adventitious shoot regeneration by light (**Figure 2B**). In *Arabidopsis* ‘col’, it was observed that *phyA* mutant explants produced more adventitious shoots compared to the wild type, while the numbers of adventitious shoots were drastically reduced in *phyB* and *cry1/cry2*. These results suggest that PHYA inhibits adventitious shoot regeneration, whereas PHYB and CRY1/2 promote adventitious shoot regeneration. Previous studies have shown that PHYA/B and CRY1/2 inhibited COP1 activity and stabilized HY5 under blue and red light, respectively (Lu et al., 2015; Zuo et al., 2011). In the *hy5-215* mutant, the number of adventitious shoots regenerated from the roots was significantly higher than in the wild type (Dai et al., 2022). Given that the phenotypes of *cry1/cry2* and *phyB* mutants were opposite to those of *hy5-215*, these findings suggest that CRY1/2 and PHYB regulate adventitious shoot regeneration through multiple pathways, with the facilitative pathway dominating, further highlighting the complexity of light regulation in this process. In darkness, COP1 binds to HY5 to suppress its activity, while HY5 mediates light signaling under light (Xu, 2020). In *Arabidopsis*, the rate of adventitious shoot regeneration was significantly lower in the *cop1* mutant and higher in the *hy5* mutant compared with the wild type. HY5 is directly bound to the promoters of *WUS* and *CLV3* to inhibit their expression, thereby suppressing adventitious shoot regeneration (Dai et al., 2022). Additionally, *HY5* is also bound to the promoter of *ARR12* to inhibit its expression, while ARR12 directly promotes *WUS* expression. Therefore, *HY5* inhibits adventitious shoot regeneration through multiple pathways by downregulating downstream *WUS* expression. In the immature embryos of the barley ‘K3’, the expression of *9-CIS-EPOXYCAROTENOID DIOXYGENASE 1* (*NCED1*), an enzyme involved in abscisic acid (ABA) biosynthesis, was downregulated under darkness, leading to reduced ABA synthesis. Exogenous ABA, in turn, inhibited the regeneration of callus and adventitious shoots. This suggests that light also influences adventitious shoot regeneration by regulating both auxin and abscisic acid biosynthesis (Rikiishi et al., 2015).

## 3 Somatic embryogenesis

### 3.1 Basic process and molecular mechanisms of somatic embryogenesis

Somatic embryogenesis can be classified as either direct (**Figure 1E**) or indirect, depending on whether embryonic callus is formed. Indirect somatic embryogenesis is the predominant form and involves three main stages (**Figure 1F**). First, somatic cells dedifferentiate to form callus; then, the callus acquires pluripotency and is capable of further differentiation; finally, the embryonic callus regenerates somatic embryos (Halperin, 1966; Raghavan, 2004). Indirect somatic embryogenesis has a higher propagation coefficient and is more effective for the conservation of valuable germplasm resources (Yang and Zhang, 2010). Numerous factors affect somatic embryogenesis, with explant type, the developmental stage of the mother plant, and auxin being among the most important factors (Wang et al., 2020). For example, the addition of 2,4-D promoted somatic embryogenesis in *Arabidopsis* by inhibiting the exocytosis of endogenous auxin (Karami et al., 2023). Auxin, in turn, further promoted somatic embryogenesis by activating the expression of cellular totipotency factors (Braybrook et al., 2006). In addition, various abiotic stresses also play a role in inducing somatic embryogenesis. For instance, desiccation treatment with PEG in the medium promoted somatic embryogenesis in *Picea asperata* and *Cunninghamia lanceolata* (Jing et al., 2017; Zhou et al., 2017). The addition of sucrose to the culture medium, as well as exposure to low or high temperatures and heavy metals treatment, has also been shown to be useful for the induction of somatic embryogenesis (Gao et al., 2022; Miroshnichenko et al., 2013; Fehér, 2015).

Somatic embryogenesis is regulated by several key transcription factors (**Figure 1F**), including WUS, BABY BOOM (BBM), LEAFY COTYLEDON 1/2 (LEC1/2), ABSCISIC ACID INSENSITIVE 3 (ABI3), and FUSCA 3 (FUS3) and AGAMOUS-LIKE 15 (AGL15), whose roles are conserved across plants (**Figure 1**). Most of these factors were induced by auxin (Horstman et al., 2017) and, in turn, promoted the synthesis of endogenous auxin. For example, BBM directly upregulated the expression of the auxin synthesis gene *YUCCA 3/8* (*YUC3/8*) in *Arabidopsis* (Li et al., 2022a), and LEC2 activated the expression of *YUC2* and *YUC4* to promote auxin synthesis (Stone et al., 2008). Additionally, there is mutual regulation among these key factors. BBM transcriptionally regulated *LEC1* and *LEC2*, as well as the two other LAFL genes, *FUS3* and *ABI3* (Horstman et al., 2017). *WOX2* was strongly expressed during somatic embryogenesis in *Arabidopsis* overexpressing *LEC2*, compared to the wild type. CHIP-seq data showed that LEC2 is directly bound to the promoter of *WOX2*, promoting its expression (Wang et al., 2020). The expression of *LEC2* and *ABI3* was increased in *35Spro: AGL15* seeds (Braybrook et al., 2006; Zheng et al., 2009). AGL15 also activated the expression of *SOMATIC EMBRYOGENESIS RECEPTOR-LIKE KINASE1* (*SERK1*) (Kwaaitaal and De Vries, 2007). Moreover, both AGL15 and FUS3 regulated the expression of *Gibberellin 2-oxidase 6* (*GA2ox6*) to regulate gibberellin content in *Arabidopsis* and *Glycine*, thereby influencing somatic embryogenesis (Wang et al., 2004; Zheng et al., 2016). Epigenetic regulation also plays a role in somatic embryogenesis. For example, trichostatin A (TSA), an inhibitor of histone deacetylase (HDA), induced somatic embryogenesis in cotyledon explants of *Arabidopsis* in the absence of exogenous auxin, while significantly reducing HDA activity (Wójcikowska et al., 2018). The DNA methylation inhibitor 5-azacytidine (5-Aza-C) inhibited the formation of embryonic cell clusters in epidermal carrot cells and downregulated the expression of *LEC1* during somatic embryogenesis in carrot (Yamamoto et al., 2005).

### 3.2 Effect of light on somatic embryogenesis

#### 3.2.1 Light intensity and light spectra

Light intensity significantly affects somatic embryogenesis (**Table 2**). In *Aralia elata* Miq., the induction rate of somatic embryos reached 88.89% under 2,000 lux (37.04 μmol·m^−2^·s^−1^). As the light intensity increased, the induction rate decreased, indicating that an optimal light intensity is beneficial for somatic embryo formation (Cheng et al., 2021b). Light promoted somatic embryogenesis from spinach root sections (Milojević et al., 2012). The number of SEs increased significantly with light intensity from 0 to 100 μmol·m^−2^·s^−1^ and then decreased at 150 μmol·m^−2^·s^−1^, and the regeneration of SEs started 4 weeks earlier in explants cultured at 100 μmol·m^−2^·s^−1^ than at 150 μmol·m^−2^·s^−1^ or in the dark (Milojević et al., 2012). More studies have focused on the effects of light spectra and photoperiod. Red light, in particular, promoted somatic embryogenesis in various plant species. Under red light, the embryonic callus of *Rosa chinensis* Jacq. produced more somatic embryos (Chen et al., 2014). This was because the callus under red light turned reddish-brown and retained its ability to continuously generate embryos, while the callus under white light hardened and lost its embryogenic potential during subculture. Red light enhances cytokinin levels, maintaining hormone balance and promoting somatic embryo induction. Under red light, the somatic embryo induction rate of *Dactylorhiza umberosa* protocorm explants reached 95%, with 25 primary embryos formed (Naderi Boldaji et al., 2023). Among all spectra, the explant seeds of *Ajuga bracteosa* under red light exhibited the highest DPPH-radical scavenging activity, reaching 92.86% (Rukh et al., 2019). Under red light, auxin production increased and redox balance was maintained in the shoots of *Begonia × tuberhybrida* Voss and the hypocotyls of *Gossypium hirsutum* L., which supported the preservation of embryonic callus and promoted somatic embryo formation (Van The Vinh et al., 2023; Yu et al., 2019). Similarly, leaf explants of *Chrysanthemum* showed higher somatic embryo induction rates under red light (Hesami et al., 2019).

**Table 2:** The effect of light on somatic embryogenesis in plants.

The properties of light		Species	Function	Reference
Light intensity	2,000 lx (37.04 μmol·m^−2^·s^−1^·m^−2^·s^−1^)	*Aralia elata* (Miq.) Seem	Improvement of induction rate and number of somatic embryos	(Cheng et al., 2021b)
Light spectra	Red	*Gossypium hirsutum* L.	Promoting the formation and proliferation of embryogenic callus	(Yu et al., 2019)
	Red	*Begonia × tuberhybrida* Voss	Improvement the number of somatic embryos	(Van The Vinh et al., 2023)
	Red or white	*Epipactis veratifolia*	Improvement of induction rate and number of direct somatic embryogenesis	(Naderi Boldaji et al., 2023)
	White	*Dactylorhiza umberosa*	Improvement of induction rate of direct somatic embryogenesis	(Naderi Boldaji et al., 2023)
	Red or blue	*Dianthus caryophyllus*	Promotion the formation of embryogenic callus andnumber of somatic embryos	(Aalifar et al., 2019)
	Far-red or green	*D. caryophyllus*	Reduction the formation of embryogenic callus andnumber of somatic embryos	(Aalifar et al., 2019)
	White and blue	*Carica papaya* L.	Improvement the number somatic embryos	(Almeida et al., 2019)
	Red or blue light	*Chrysanthemum*	Improvement/reduction of induction rate of somatic embryos	(Hesami et al., 2019)
	Red or blue	*Ajuga bracteosa*	Improvement/reduction of induction rate of somatic embryos	(Rukh et al., 2019)
	White, red, and far-red	*Saccharum* spp.	Improvement the number of somatic embryos	(Heringer et al., 2017)
	White	*Abies nordmanniana*	Improvement of induction rate of somatic embryos	(Nawrot-Chorabik, 2016)
	Red and blue	*Peucedanum japonicum* Thunb.	Improvement of induction rate of somatic embryos	(Chen et al., 2016a)
	Yellow or red	*Panax vietnamensis* Ha *et* Grushv.	Promotion/reduction the formation of embryogenic callus	(Nhut et al., 2015)
	Red and blue	*P. vietnamensis* Ha *et* Grushv.	Promotion the maturation of somatic embryos	(Nhut et al., 2015)
	Red	*Rosa chinensis* Jacq.	Improvement the number of somatic embryos	(Chen et al., 2014)
	Red and far-red	*Doritaenopsis*	Improvement the number of somatic embryos	(Park et al., 2010)
Photoperiod	Darkness for 5 weeks	*Lycium barbarum* L.	Promoting the formation of embryogenic callus	(Khatri and Joshee, 2024)
	Darkness	*Rhynchostylis gigantea*	Improvement the number of somatic embryos	(Rianawati et al., 2023)
	14/10 h (light/dark)	*Ginkgo biloba*	Promotion the maturation of somatic embryos	(Chen et al., 2023)
	16/8 h (light/dark)	*Olea europaea* L.	Promotion the germination of somatic embryos	(Mazri et al., 2020)
	16/8 h (light/dark)	*Spinacia oleracea* L.	Promotion the induction of somatic embryos	(Belić et al., 2020)
	16/8 h (light/dark)	*Pistacia vera* L.	Improvement the number of somatic embryos	(Ghadirzadeh-Khorzoghi et al., 2019)
	Darkness	*Typha domingensis*	Promotion the maturation of somatic embryos	(Hernández-Piedra et al., 2018)
	Darkness	*Epipactis veratrifolia*	Improvement of induction rate of somatic embryos	(Moradi et al., 2017)
	16/8 h (light/dark)	*Cyathea delgadii* Sternb.	Improvement of induction rate of somatic embryos	(Mikuła et al., 2015)
	Darkness for 2 weeks	*Campanula punctata* Lam. var. *rubriflora* Makino	Improvement of induction rate of somatic embryos	(Sivanesan et al., 2011)
	16/8 h (light/dark)	*Lilium ledebourii* (Baker) Bioss.	Increasing the proportion of embryogenic callus	(Bakhshaie et al., 2010)
	16/8 h (light/dark)	*Cinnamomum camphora* L.	Improvement the number of somatic embryos	(Shi et al., 2009)
	16/8 h (light/dark)	*Cucumis sativus*	Increasing the weight of somatic embryos	(Elmeer and Hennerty, 2008)
	Darkness	*Fragaria* sp.	Improvement of induction rate of somatic embryos	(Biswas et al., 2007)
	Darkness	*Eucalyptus globulus* Labill	Promoting the formation of embryogenic callus	(Pinto et al., 2008)

In addition to red light, it has been observed that the combination of red light with other wavelengths also promotes somatic embryogenesis. Under mixed red and blue light, root explants of *Peucedanum japonicum* Thunb. produced the highest number of somatic embryos, with a better effect than red or blue light alone (Chen et al., 2016a). In *Panax vietnamensis* Ha *et* Grushv, the highest rate of somatic seedling formation from embryonic callus was achieved under a combination of 60% red and 40% blue light (Nhut et al., 2015). Furthermore, the combination of red and far-red light induced the highest number of somatic embryos in *Doritaenopsis* inflorescence explants while maintaining a low level of endoreduplication (Park et al., 2010). In addition, under red and blue light, an average of 58 somatic embryos were produced per callus, significantly higher than the 23 embryos generated under fluorescent light (Heringer et al., 2017). Proteomic analysis of callus treated with different light spectra revealed a 23-fold increase in the expression of the methyltransferase *PROBABLE METHYLTRANSFERASE 19-LIKE* (*pmt19-like*). These results suggest that protein methylation also plays a role in the response to mixed light spectra.

Blue, green, far-red, and white light also influence somatic embryogenesis in plant species. In *A. bracteosa*, leaf explants were unable to produce somatic embryos under blue light, likely due to an increase in phenolic compounds that inhibited the differentiation of embryonic callus into somatic embryos (Rukh et al., 2019). Blue light promoted the maturation of somatic embryogenesis in radiata pine, and the plant height of somatic embryo plants was significantly increased after blue light treatment (Castander-Olarieta et al., 2023). Both green and far-red light inhibited the formation of embryonic callus in *Dianthus caryophyllus* (Aalifar et al., 2019). The embryonic callus of *Abies nordmanniana* produced the highest number of somatic embryos under white light compared to blue and far-red light (Nawrot-Chorabik, 2016). Proteomic analysis revealed increased abundance of proteins associated with energy production, such as ALCOHOL DEHYDROGENASE 1 (ADH1), GLYCERALDEHYDE-3-PHOSPHATE DEHYDROGENASE (GAPDH), and TRIOSE PHOSPHATE ISOMERASE (TPI), as well as proteins related to the cell wall, including PEPTIDOGLYCAN (PG) and GERMIN-LIKE PROTEINS (GLPs) (Almeida et al., 2019). White light promoted somatic embryogenesis in *Carica papaya* L. by affecting processes such as energy production and cell wall synthesis (Almeida et al., 2019). In summary, light spectra affect the efficiency of somatic embryogenesis in plants primarily by modulating hormone levels, redox balance, phenolic production, and cell division.

#### 3.2.2 Photoperiod

Photoperiod influences the induction of somatic embryogenesis (**Table 2**). Many plant explants regenerate more somatic embryos in darkness. Leaves of *Rhynchostylis gigantea* incubated in darkness for 3 weeks produced more somatic embryos compared to those cultivated under a 16/8-h photoperiod, with induction rates of 93.8% and 77.1%, respectively (Rianawati et al., 2023). Leaf explants of *Lycium barbarum* L. were more easily induced to form embryonic callus and somatic embryos when cultured in darkness for 5 weeks (Khatri and Joshee, 2024). In *Campanula punctata* Lam. var. *rubriflora*, somatic embryos were successfully regenerated from leaf and petiole explants under both darkness for 2 weeks and a 16/8-h photoperiod, with higher efficiency observed in darkness (Sivanesan et al., 2011). Similarly, different photoperiodic treatments, ranging from 24-h light to 24-h darkness, were tested for somatic embryo induction in the embryonic callus of *Fragaria* sp. The results showed that 24-h darkness was the optimal photoperiod for somatic embryo induction, while exposure to more than 6 h of light per day reduced somatic embryo induction in strawberries (Biswas et al., 2007). A higher number of somatic embryos was also observed under an initial 24-h dark treatment compared to the 16/8-h photoperiod in *Eucalyptus globulus* and *Epipactis veratrifolia* (Moradi et al., 2017; Pinto et al., 2008).

Explants of some plant species produce more somatic embryos under photoperiods with longer light durations. For example, the somatic embryo induction rate of *Ginkgo biloba* was higher under a 14/10-h photoperiod (light/dark) than in darkness, and gibberellic acid (GA_3_) levels were elevated. RNA-seq data revealed that genes related to photosynthesis and carbon fixation, such as *Psb A* and *Psb C*, were significantly upregulated under a 14/10-h photoperiod (Chen et al., 2023). Similarly, under a 16/8-h photoperiod, the somatic embryogenesis induction efficiency of *Olea europaea* L. seeds reached 45%, which was higher than the 35% observed in darkness. Furthermore, the regeneration rate of adventitious shoots from somatic embryos was only 5% in darkness, significantly lower than the 45% observed under the 16/8-h photoperiod (Mazri et al., 2020). A similar pattern was found in the somatic embryogenesis of *Spinacia oleracea* L. Genes related to the synthesis of GA_3_, such as *GA20-ox1* and *GA3-ox1*, were highly expressed under 16/8-h photoperiod, indicating that light regulated somatic embryogenesis by modulating the level of GA_3_ (Zdravković-Korać et al., 2023). Immature syncytial explants of *Pistacia vera* L. showed browning and produced fewer somatic embryos when cultured in darkness compared to 16/8-h photoperiod (Ghadirzadeh-Khorzoghi et al., 2019). Additionally, more somatic embryos were induced in *Cinnamomum camphora* L. and *Cyathea delgadii* Sternb. at 16/8-h photoperiod compared to darkness (Mikuła et al., 2015; Shi et al., 2009). In summary, photoperiod regulates somatic embryogenesis in plant species by influencing the callus state, photosynthesis, carbon fixation, and gibberellin synthesis.

#### 3.2.3 Molecular mechanisms of light-regulated somatic embryogenesis

**Figure 3:**
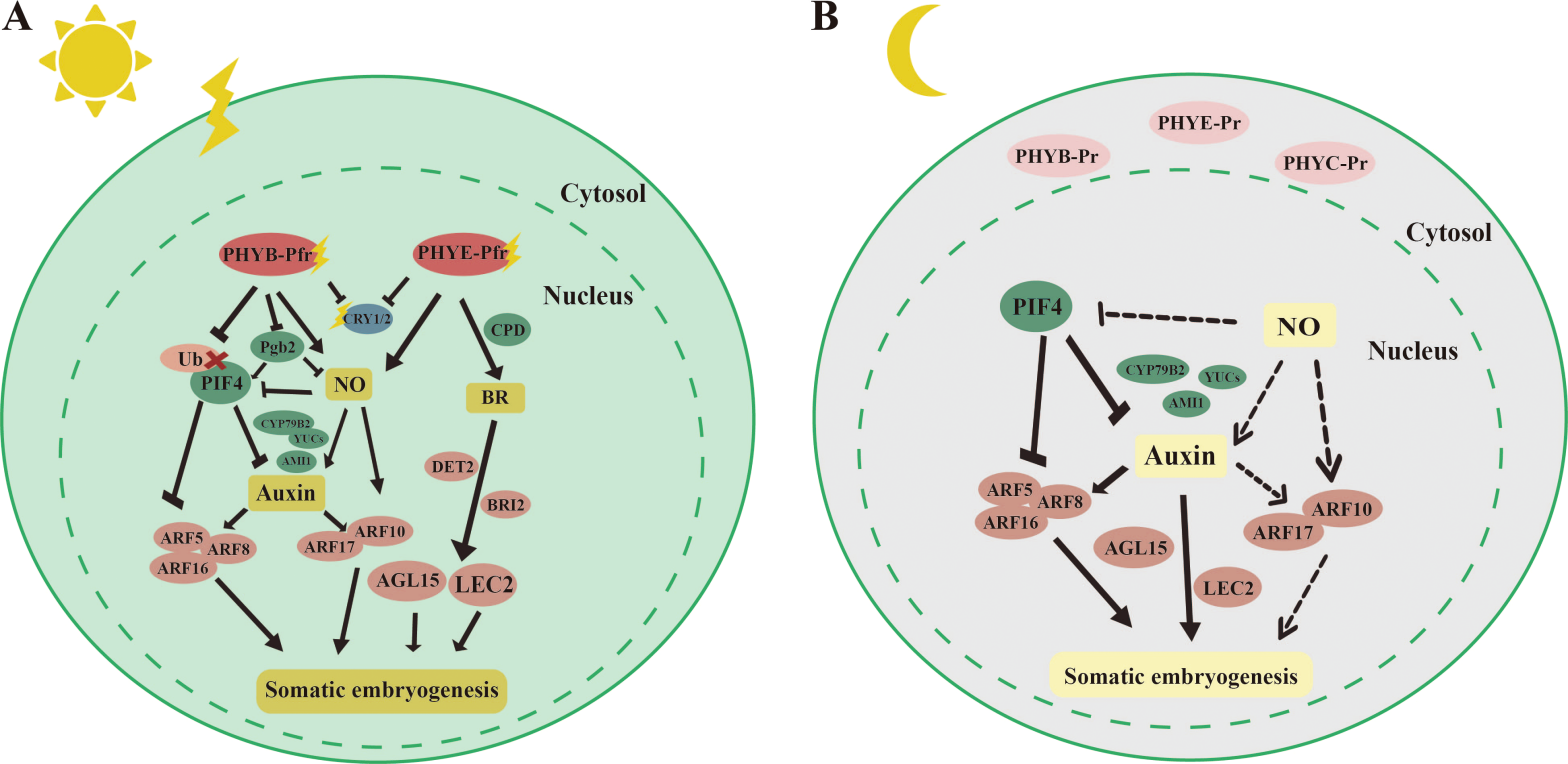
Molecular mechanisms by which light promotes somatic embryogenesis. [**(A)**, left] *Phytochrome Interacting Factor 4* (*PIF4*) can inhibit somatic embryogenesis by suppressing the expression of auxin synthesis genes *Cytochrome P450*, *family 79*, *subfamily B*, *polypeptide 2* (*CYP79B2*), *YUCCA*s (*YUC*s), and *AMI1*, as well as auxin response factors *ARF5*, *ARF8*, and *ARF12*. Under light, PHYA can target PIF4 for degradation. PHYB and PHYE can activate downstream NO, which in turn upregulates the expression of auxin synthesis genes *YUC*s and *AMI1*, as well as auxin response factors *ARF10/17*, to promote somatic embryogenesis. PHYE can also promote the accumulation of brassinosteroids (BRs) by activating the expression of the BR synthesis gene *CONSTITUTIVE PHOTOMORPHOGENESIS AND DWARF 3* (*CPD3*). BRs, in turn, promote somatic embryogenesis by activating the expression of downstream *AGAMOUS-LIKE 15* (*AGL15*) and *LEAFY COTYLEDON 2* (*LEC2*). [**(B)**, right] Under dark conditions, phytochromes PHYB, PHYE, and PHYC exist in an inactive form in the cytoplasm, while *PIF4* is expressed in the nucleus. The level of NO in the nucleus is low. *PIF4* can inhibit somatic embryogenesis by suppressing the expression of auxin synthesis genes *CYP79B2*, *YUC*s, and *AMI1*, as well as auxin response factors *AUXIN RESPONSE FACTOR 5* (*ARF5*), *ARF8*, and *ARF12*. The straight arrow represents activation, the connection of the blunt end represents suppression, parallel lines indicate interactions, and dashed lines indicate non-functionality under certain conditions.

We focus on the molecular mechanisms by which light promotes somatic embryogenesis in plant species (**Figure 3**). In *Arabidopsis*, immature zygotic embryos produced more embryonic callus and somatic embryos when exposed to light. Under light, both PHYB and PHYE may inhibit CRY1/2-mediated blue light signaling (Chan and Stasolla, 2023). Mutants of *phyB* and *phyE* exhibited significantly lower somatic embryogenesis efficiency compared to the wild type, while *phyC* mutants showed higher levels of somatic embryogenesis. These results suggest that PHYB and PHYE promote somatic embryogenesis, whereas PHYC inhibits this process (Chan and Stasolla, 2023), highlighting the complexity of light signaling in regulating somatic embryo formation. Under light (**Figure 3A**), PHYB targeted PIF4 for degradation, alleviating the inhibitory effect of PIF4 on auxin synthesis and signaling (Mira et al., 2023). PHYB and PHYE translocated to the cell nucleus, where they activated the production of downstream nitric oxide (NO), a small gaseous molecule known to be involved in light signaling in *C. melo* L (Melo et al., 2016). The accumulation of NO increased the auxin maxima at the origin of callus formation in *Arabidopsis*. This effect was mediated by NO upregulating the expression of auxin synthesis genes such as *YUC*s and *AMI1*, as well as the transcription factors *ARF10* and *ARF17* (Elhiti et al., 2013). Endogenous auxin directly regulated *BBM* and *LEC1/2* to promote somatic embryogenesis (Weijers and Wagner, 2016). The addition of NO was found to elevate the expression of *AGL15*, although the exact mechanism by which NO regulated *AGL15* remained unclear (Chan and Stasolla, 2023). Moreover, PHYE promoted somatic embryogenesis by increasing the content of brassinosteroids (BRs). It achieved this by activating *CONSTITUTIVE PHOTOMORPHOGENESIS AND DWARF 3* (*CPD3*), a gene involved in BR synthesis. BRs, in turn, promoted somatic embryogenesis by enhancing the transcription of downstream genes *AGL15* and *LEC2*. Furthermore, the number of somatic embryos was reduced in *det2* and *bri2* mutants, key factors in the BR signaling pathway (Chan and Stasolla, 2023). Furthermore, under dark conditions (**Figure 3B**), the phytochromes PHYB, PHYE, and PHYC remained inactive in the cytoplasm, resulting in low levels of NO in the nucleus, which weakened the promotive effect of NO on somatic embryogenesis. In this context, PIF4, which was active in the nucleus, played a key role in regulating somatic embryogenesis (Cheng et al., 2021a). In *pif4* mutants, genes involved in auxin biosynthesis, such as *AMI1*, *YUC*s, and *Cytochrome P450* (*CYP79B2*), as well as transcription factors related to auxin signaling, including ARF5/8/16, were upregulated (Mira et al., 2023). Consequently, PIF4 inhibits somatic embryogenesis by repressing both auxin synthesis and signaling pathways.

## 4 Adventitious root regeneration

### 4.1 Basic process and molecular mechanisms of adventitious root regeneration

There are several ways to regenerate AR, with this discussion focusing on AR regenerated from cuttings, *de novo* root organogenesis, and HAR. Plant cuttings involve inserting isolated plant leaves or stem segments into soil, sand, or water, allowing them to root and form a complete plant. *De novo* root organogenesis refers to the regeneration of AR from callus tissue formed at a damaged site of an isolated explant (**Figure 1D**). HARs, however, are induced from the hypocotyls of plants under various stress conditions (**Figure 1G**). A classic model for studying *de novo* root organogenesis is the formation of AR from isolated *Arabidopsis* leaves on a medium without added plant growth regulators (Verstraeten et al., 2013). This process is generally divided into three stages: first, isolated explants, such as leaves and sense wound signals; second, specific cells in the explant respond to these signals by synthesizing auxin and transporting it to stem cells (e.g., the formation layer near the wound); and finally, ARs are induced from these stem cells in the presence of auxin (Xu, 2018). HARs are also produced during plant growth and development in response to environmental stresses. For example, under flooding stress, HAR in *Cucumis sativus* L. improved gas exchange and nutrient uptake, compensating for the loss of primary roots (Pan et al., 2024). When seeds of *Arabidopsis* were incubated in the dark for 3 days and then transferred to blue light, the hypocotyls induced HAR (Zeng et al., 2022).

During *de novo* root organogenesis, isolated explants receive transient wound signals mediated by H_2_O_2_, ROS, JA, and ethylene (**Figure 1D**) (Liu W. et al., 2022; Yuan et al., 2024). These signals triggered auxin synthesis and accumulation at specific sites in *Arabidopsis*, with auxin then transported to the vicinity of the wound (Zhang et al., 2019). Auxin gradually accumulated in the stem cells of the vascular cambium, preparing for subsequent AR regeneration. At the wound site, auxin activated the expression of *WOX11*, which signified a shift in cell fate and marked the initiation of root primordium formation (Liu et al., 2014). WOX11 formed a complex with ARF6/8, which in turn activated the expression of downstream genes such as *LBD16* and *RGF1INSENSITIVE 1* (*RGI1*) (Hu and Xu, 2016; Zhang et al., 2023). Simultaneously, WOX11, along with PLT3/5/7, activated the expression of *WOX5*. PLT3/5/7 also promoted the expression of *PLT1/2*, which facilitated cell division and the formation of the root meristem (Liu J. et al., 2022; Xu, 2018). The formation of HAR also requires auxin involvement. *ARF7/19*, which were implicated in lateral root (LR) formation, were similarly involved in HAR formation (Lee et al., 2019). Additionally, ARF6/8/17 and the auxin signaling components TIR1/AFB2 (AUXIN-SIGNALING F-BOX 2) were key regulators in HAR formation (Gutierrez et al., 2012, 2009; Lakehal et al., 2019; Sorin et al., 2005).

### 4.2 Effect of light on adventitious root regeneration

#### 4.2.1 Light intensity

Light intensity plays a significant role in *de novo* root organogenesis (**Table 3**). Studies have shown that in *Arabidopsis*, during AR regeneration from cotyledons, increasing light intensity significantly reduced AR formation efficiency (Blair Nameth et al., 2018). Under higher light intensity, the levels of ROS increased, while the content of the photoprotective pigment zeaxanthin decreased in the explants. This imbalance led to photo-oxidative damage, which further impaired AR regeneration. In *Prunus serotina*, AR regeneration from axillary buds was studied under five light-intensity gradients ranging from 0 to 833 μmol·m^−2^·s^−1^. Results indicated a negative correlation between light intensity and AR formation, with the highest number of ARs observed at light intensities of 0 and 70 μmol·m^−2^·s^−1^ (Fuernkranz et al., 1990). Similarly, cuttings of *Pisum sativum* formed more ARs under 16 W·m^−2^ (31.37 μmol·m^−2^·s^−1^) than under 38 W·m^−2^ (74.51 μmol·m^−2^·s^−1^) (Hansen, 1975). Overall, higher light intensity inhibited *de novo* root organogenesis by disrupting the redox balance in the explants, leading to photo-oxidative damage. In contrast, light enhanced HAR formation in *Nelumbo nucifera* Gaertn. (Cheng et al., 2020). HARs in seedlings were induced by light, with faster formation observed as light intensity increased. RNA-seq analysis revealed that genes related to auxin (IAA) synthesis and carbohydrate metabolism were highly expressed under high light intensity, suggesting that light promotes HAR formation by influencing both auxin synthesis and photosynthesis (Cheng et al., 2020).

**Table 3:** The effect of light on adventitious root regeneration in plants.

The properties of light		Species	Function	Reference
Light intensity	20–25 μmol·m^−2^·s^−1^	*Arabidopsis thaliana*	Promoting regeneration of adventitious root (AR) from explant leaves	(Blair Nameth et al., 2018)
	0–70 μmol·m^−2^·s^−1^	*Prunus serotina*	Promoting regeneration of AR from axillary shoots	(Fuernkranz et al., 1990)
	16 W·m^−2^ (31.37 μmol·m^−2^·s^−1^)	*Pisum sativum* L.	Promoting regeneration of AR from cuttings	(Hansen, 1975)
	5,000–20,000 lx (208.33–370.37 μmol·m^−2^·s^−1^)	*Nelumbo nucifera* Gaertn.	Accelerating the development of hypocotyl adventitious root (HAR)	(Libao et al., 2020)
Light spectra	Red and blue	*Gerbera jamesonii* cv.	Promoting regeneration of AR from shoot tips	(Lim et al., 2023)
	Far-red	*Citrullus lanatus*	Promoting regeneration of AR from rootstock	(Wu et al., 2023)
	Red	*Camellia gymnogyna* Chang	Improvement of regeneration rate of AR from tissue culture seedlings	(Fu et al., 2023)
	Blue or red	*Camellia sinensis*	Promoting/inhibiting regeneration of AR from cuttings	(Shen et al., 2022)
	Blue	*A. thaliana*	Promoting regeneration of HAR	(Zeng et al., 2022; Zhai et al., 2021)
	Red, blue, purple, and green	*Cunninghamia lanceolata*	Improvement of regeneration rate of AR from tissue culture seedlings	(Xu et al., 2020)
	Blue	*Chrysanthemum*	Promoting regeneration of AR from leaf-bud cutting	(Gil et al., 2020)
	Red	*Picea abies*	Promoting regeneration of AR from cutting	(Alallaq et al., 2020)
	White	*Wikstroemia gemmata*	Promoting regeneration of AR from stem	(Verstraeten et al., 2020)
	Low red:far-red ratio	*Chrysanthemum morifolium*	Promoting regeneration of AR from unrooted cuttings	(Christiaens et al., 2019)
	Red or blue	*Phaseolus vulgaris* L.	Promoting/inhibiting regeneration of AR from hypocotyl segment	(Fletcher et al., 1965)
	Yellow or blue	*P. serotina*	Promoting/inhibiting regeneration of AR from axillary shoots	(Fuernkranz et al., 1990)
	Red or blue	*Morinda citrifolia*	Promoting/inhibiting regeneration of AR from leaf explants	(Baque et al., 2010)
	Red and blue	*Prunus avium* L. × *Prunus cerasus L.*	Promoting regeneration of AR from rootstock	(Iacona and Muleo, 2010)
Photoperiod	Darkness	Grapevine (*Vitis* sp.)	Promoting regeneration of AR from leave-petiole	(Yuan et al., 2024)
	Darkness	*A. thaliana*	Promoting regeneration of HAR	(Li et al., 2021)
	16/8 h (light/dark)	*C. lanceolata*	Promoting regeneration of HAR from tissue culture seedlings	(Xu et al., 2020)
	Darkness	*Eucalyptus globulus*	Promoting regeneration of HAR from epicotyl	(Fett-Neto et al., 2001)
	16/8 h (light/dark)	*Betula pendula*	Promoting regeneration of HAR from stem segment	(Wynne and McDonald, 2002)
	Darkness for 10 d	*Petunia × hybrida*	Promoting regeneration of AR from cutting	(Klopotek et al., 2010)
	Darkness for 4 weeks	*Dianthus caryophyllus*	Promoting regeneration of AR from cutting	(Agulló-Antón et al., 2011)
	16/8 h (light/dark)	*Linum usitatissimum* L.	Promoting regeneration of AR from hypocotyl	(Siegień et al., 2013)
	12/12 h (light/dark)	*Corylus avellana* L.	Promoting regeneration of AR from cutting	(Tombesi et al., 2015)

#### 4.2.2 Light spectra

Light spectra significantly influence AR regeneration (**Table 3**). Studies have shown that mixed light promotes AR regeneration. For example, compared to red or blue light, *Prunus avium* L. × *Prunus cerasus* L. cuttings regenerated more ARs and produced longer roots under mixed red and blue light, indicating a synergistic effect between the two photoreceptors (Iacona and Muleo, 2010). Similarly, mixed red and blue light enhanced AR formation in *Gerbera jamesonii* (Lim et al., 2023). Explants under this light combination exhibited the highest photosynthetic rate, internal CO_2_ concentration, and stomatal conductance, which in turn promoted AR regeneration by improving photosynthetic efficiency and respiration. The addition of violet and green light to red and blue light also promoted AR regeneration in *C. lanceolata* cuttings (Xu et al., 2020). Under this mixed light, explants showed the highest levels of chlorophyll *a*, chlorophyll *b*, and total chlorophyll, along with improved maximum quantum yield of PSII (Fv/Fm), photochemical quenching coefficient (qp), and relative electron transport rate in PSII (ETRII). Stepwise regression analysis revealed significant correlations between Fv/Fm, qp, ETRII, and AR formation. Furthermore, the addition of NPA reduced AR formation in *Chrysanthemum* cuttings (Christiaens et al., 2019), but lower ratios of red to far-red light partially rescued this inhibitory effect, suggesting that a mix of red and far-red light promotes AR regeneration by influencing auxin polar transport.

Red and blue light have significant effects on AR formation. Red light promoted AR regeneration in *Camellia gymnogyna* Chang cuttings (Fu et al., 2023). RNA-seq data revealed that genes highly expressed under red light were enriched in pathways related to auxin and hormone responses, indicating that red light regulates AR regeneration through complex hormonal interactions. In contrast, the exogenous application of JA inhibited AR regeneration in *Picea abies* (Alallaq et al., 2020). Red light facilitated AR formation by reducing JA accumulation in *P. abies* cuttings. Additionally, red light affected cell number and size, promoting AR regeneration in isolated hypocotyls of *Phaseolus vulgaris* L (Fletcher et al., 1965). In *Camellia sinensis* L. cuttings, blue light increased both the number and length of ARs (Shen et al., 2022). RNA-seq analysis showed that genes related to auxins, such as *YUC*s, *ARF*s, *AUX1*, *PIN1*, and *PIN3*, were highly expressed under blue light, suggesting that blue light regulates AR regeneration through auxin synthesis and signaling (Shen et al., 2022). The levels of ABA and *trans*-zeatin (tZ) were also higher under blue light, indicating that multiple hormones are involved in blue light-mediated AR formation in *C. sinensis* L. *Chrysanthemum* cuttings showed the highest number of ARs under blue light compared to white and red light, with *LBD1* being significantly more highly expressed under blue light (Gil et al., 2020). Furthermore, blue light facilitated the formation of HARs in *Arabidopsis*. The photoreceptors CRY1/2 and PHOT1/2 were involved in this process, and mutants of *cry1*, *cry2*, *phot1*, and *phot2* exhibited fewer ARs than the wild type (Zeng et al., 2022).

Red and blue light can also inhibit AR formation in some plant species. For example, blue light inhibited AR formation in isolated hypocotyls of *P. vulgaris* (Fletcher et al., 1965). Red light inhibited AR regeneration in *Camellia* cuttings, with low expression of *PILS7*, *PIN3*, and *PIN4* under red light (Shen et al., 2022). Other light wavelengths also affected AR regeneration in plant species. For example, far-red light stimulated the synthesis of auxin and carbohydrates, thereby facilitating AR regeneration in *Cannabis sativa* L. (Sae-Tang et al., 2024). In *Citrullus lanatus*, far-red light significantly upregulated auxin-related genes such as *IAA11*, *IAA17*, and *SAUR20*, resulting in the highest number of ARs (Wu et al., 2023). Yellow light increased the number of ARs in *P. serotina* (Fuernkranz et al., 1990). In summary, light spectra affect AR regeneration primarily by influencing photosynthetic and respiratory efficiency, auxin synthesis and transport, and hormone cross-talk in plant species.

#### 4.2.3 Photoperiod

Photoperiod plays an important role in AR regeneration (**Table 3**). Some plant species tend to produce more ARs under short-day photoperiods. In *Arabidopsis*, a photoperiodic gradient ranging from 24 hours of light to 24 hours of darkness was tested for HAR regeneration (Li et al., 2021). The results showed that HARs were formed in seedlings grown in darkness for more than 4 days, suggesting that light is not essential for HAR formation. Moreover, seedlings grown in darkness for 4 to 6 days produced more HARs than those grown for longer periods. This observation leads to the hypothesis that light promotes HAR formation by increasing carbon assimilates produced through photosynthesis. In grapevine (*Vitis* sp.) petiole cuttings, darkness treatment achieved a 100% rooting rate after 20 days, compared to just 10% in the control group under a 16/8-h photoperiod (Yuan et al., 2024). RNA-seq analysis revealed high expression of genes involved in cell division, such as *EXP*s, *CYCD*s, and *XTH*s, as well as auxin influx-related genes like *PIN1*, *PIN3*, and *PIN5* (Yuan et al., 2024). In *Petunia × hybrida*, cuttings produced more ARs and had a shorter rooting cycle (from 16 days to 9 days) after 7 days of darkness treatment. Dark treatment resulted in significantly lower levels of soluble sugars and starch in the leaves compared to the 16/8-h photoperiod, suggesting that darkness has promoted carbohydrate allocation to the stem base, providing energy for root development (Klopotek et al., 2010). Darkness also promoted AR regeneration of cuttings compared to a 16/8-h photoperiod in *E. globulus* and *D. caryophyllus* (Agulló-Antón et al., 2011; Fett-Neto et al., 2001).

Additionally, some plant species are better suited to produce ARs under long-day photoperiods. For example, cuttings of *Corylus avellana* L. produced more ARs under a 16/8-h photoperiod. During rooting, leaf photosynthesis provides carbohydrates necessary for AR formation (Tombesi et al., 2015). In *C. lanceolata*, three photoperiods—8/16 h (light/dark), 16/8 h (light/dark), and 24 h of light—were tested for AR regeneration (Xu et al., 2020). The results showed that explants produce the highest number of ARs at a 16/8-h photoperiod and the longest roots, along with the highest chlorophyll *a*, chlorophyll *b*, and total chlorophyll content. In *Betula pendula*, stem segments under a 16/8-h photoperiod reached 100% rooting compared to 75% in darkness (Wynne and McDonald, 2002). Similarly, under a 16/8-h photoperiod, hypocotyl explants of *L. usitatissimum* L. produced more ARs (Siegień et al., 2013). Overall, photoperiod affects the AR formation of plant species primarily by influencing photosynthesis, carbohydrate partitioning, cell division, and auxin transport.

#### 4.2.4 Molecular mechanisms of light-regulated adventitious root regeneration

**Figure 4:**
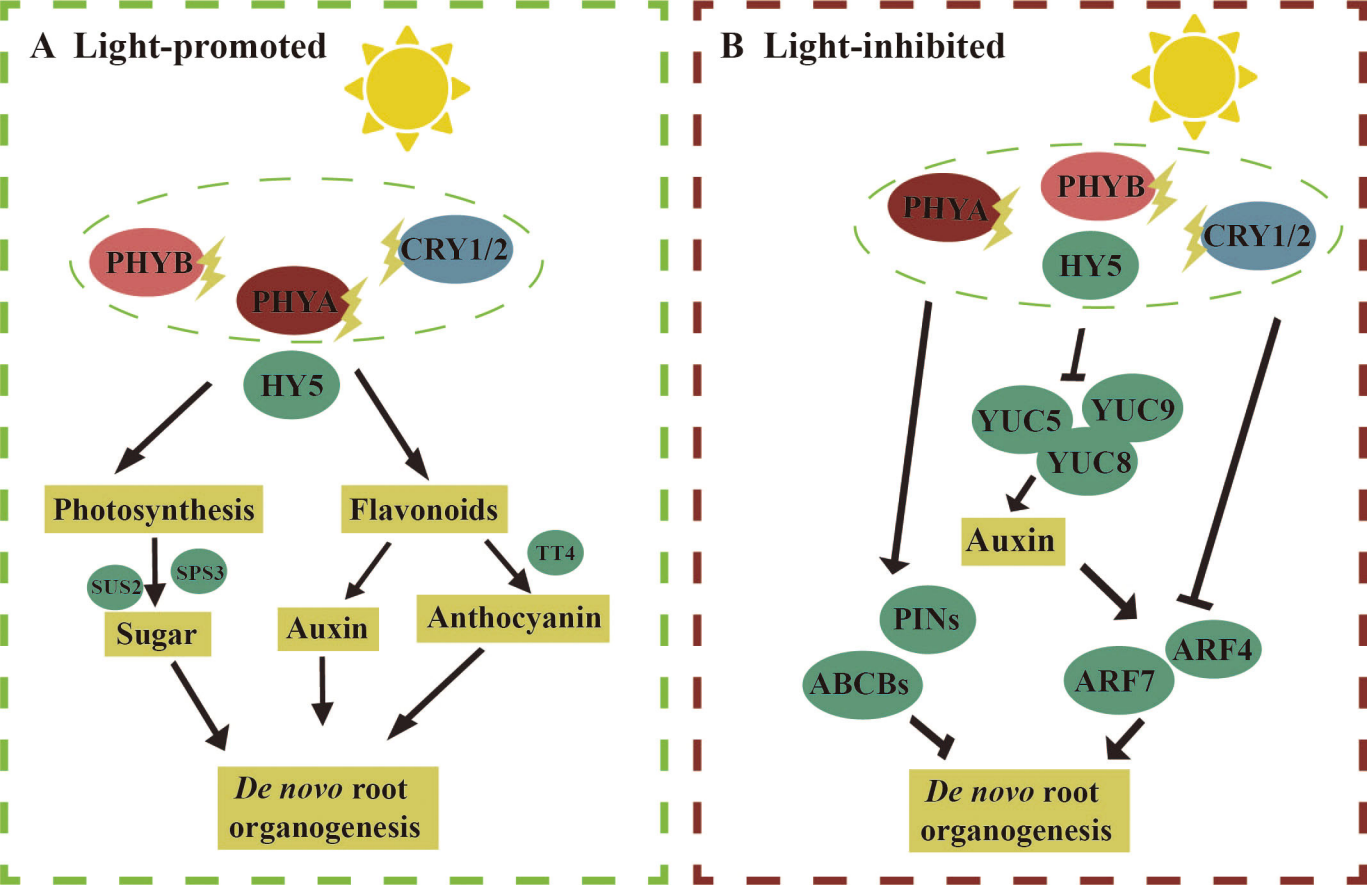
Molecular mechanism of light regulation of *de novo* root organogenesis. (**A**, left) Light promotes *de novo* root organogenesis: under light conditions, the expression levels of genes involved in sucrose synthesis, such as *Sucralose Synthase 2* (*SUS2*) and *Sucralose Phosphate Synthase 3* (*SPS3*), are elevated in grapevine petioles, leading to an increase in adventitious root regeneration. In *Arabidopsis*, the regeneration of adventitious roots is reduced in the tt4 mutant, which is deficient in anthocyanin synthesis, under light conditions. Flavonoids synthesized under light may regulate adventitious root *de novo* regeneration by modulating auxin transport. (**B**, right) Light inhibits *de novo* root organogenesis: under light conditions, the addition of NPA can promote *de novo* root regeneration, suggesting that light may inhibit adventitious root formation by regulating auxin transport proteins such as PIN-FORMED (PIN) and ATP-Binding Cassette B (ABCBs). The expression levels of auxin synthesis genes *YUC5/8/9* are reduced under light, and the expression of *ARF4/7*, involved in auxin signal transduction, is also lowered, indicating that light inhibits *de novo* root regeneration by regulating both auxin synthesis and signaling. The straight arrow represents activation, the connection of the blunt end represents suppression, and parallel lines indicate interactions.

**Figure 5:**
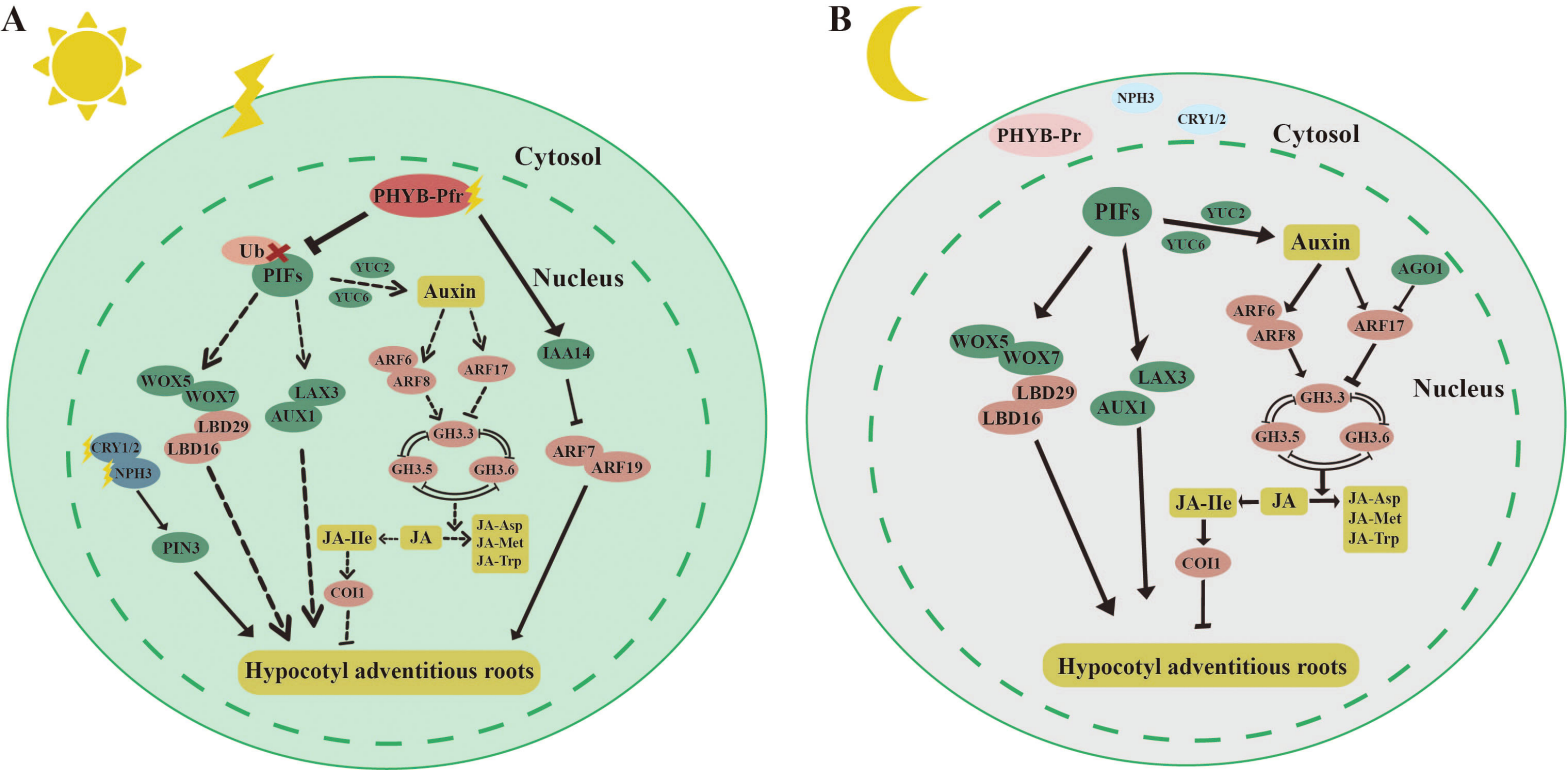
Molecular mechanisms by which light inhibits hypocotyl adventitious root (HAR). **(A)** light Under light, PHYB can target and degrade PIF4, thereby inhibiting PIF4’s suppressive effect on HAR formation. PHYB can also stabilize INDOLE-3-ACETIC ACID 14 (IAA14) through protein interactions, which lowers the expression of AUXIN RESPONSE FACTOR 7/19 (ARF7/19) and subsequently inhibits HAR formation. Blue light receptors CRY1/2, PHOTOTROPIN 1/2 (PHOT1/2), and NON-PHOTOTROPIC HYPOCOTYL 3 (NPH3) also promote the formation of *Arabidopsis* HARs by enhancing the expression of the auxin transport protein PIN3. **(B)** Under dark, PHYB-Pr exists in the inactive form in the cytoplasm, and PIFs can directly regulate key transcription factors such as LATERAL ORGAN BOUNDARY DOMAIN 16/29 (LBD16/29) and USCHEL-RELATED HOMEOBOX (WOX5/7), thereby promoting HAR formation. *PIF*s can also directly bind to the promoters of genes involved in auxin synthesis, such as *YUC2/6*, to promote HAR formation by synthesizing more auxin. Auxin response factors *ARF6* and *ARF8* can respectively upregulate and downregulate the expression of *GRETCHEN HAGEN 3.3* (*GH3.3*), *GH3.5*, and *GH3.6* to promote HAR formation. *GH3.3*, *GH3.5*, and *GH3.6* reduce the active form of jasmonic acid-lle (JA-lle) by promoting the conversion of jasmonic acid (JA) to JA-Met and JA-Asp, thereby reducing the inhibitory effect of JA on HAR through *CORONATINE-INSENSITIVE 1* (*COI1*) regulation. The straight arrow represents activation, the connection of the blunt end represents suppression, parallel lines indicate interactions, and dashed lines indicate non-functionality under certain conditions.

The pathways through which light regulates the regeneration of AR are complex. Here, we first discuss the molecular mechanisms by which light promotes and inhibits *de novo* root organogenesis (**Figure 4**). We then summarize the potential molecular mechanisms by which light inhibits HAR (**Figure 5**). To date, fewer studies have focused on the molecular mechanisms by which light promotes *de novo* root organogenesis. Light may contribute to AR formation through the synthesis of compounds such as sucrose, anthocyanins, and flavonoids (**Figure 4A**). Studies have shown that sucrose concentrations ranging from 0.5% to 2.0% were the most effective in inducing adventitious root formation in *Arabidopsis* seedlings (Takahashi et al., 2003). In grapevine (*Vitis* sp.) petiole cuttings, ARs were produced more effectively under a 16/8-h photoperiod than in darkness. Under this photoperiod, the expression of genes involved in sucrose synthesis, including *SUCROSE SYNTHASE 2* (*SUS2*) and *SUCROSE PHOSPHATE SYNTHASE 3* (*SPS3*), was elevated, suggesting that photosynthesis promotes AR regeneration in grapevine (Yuan et al., 2024). Light also promotes AR regeneration by regulating anthocyanin content. In *Arabidopsis*, the cotyledons produced more anthocyanin under light, and the regeneration efficiency of AR was significantly lower in *tt4* mutants (which have reduced anthocyanin levels) compared to the wild type (Nameth et al., 2018). Furthermore, flavonoids, which may be involved in auxin transport, have been implicated in AR formation in *Arabidopsis* (Sukumar, 2010). Under a 16/8-h photoperiod, genes involved in flavonoid synthesis, such as *CHALCONE SYNTHASE* (*CHS*) and *FLAVANONE 3-HYDROXYLASE* (*F3H*), were upregulated in grapevine (Yuan et al., 2024). However, the precise mechanisms by which these metabolites influence *de novo* root organogenesis remain to be explored in greater detail.

Next, we discuss the molecular mechanisms by which light inhibits *de novo* root organogenesis (**Figure 4B**). Under light, photoreceptors perceive light signals through photosensitive pigments such as PHYA, CRY1/2, and PHOT1/2 (Yun et al., 2023). In *Arabidopsis*, the *phyA* mutant exhibited higher efficiency of *de novo* root organogenesis compared to the wild type (Blair Nameth et al., 2018). After filtering blue light using acetate filters, cotyledon explants of *Arabidopsis* produced more ARs, suggesting that blue light may inhibit AR formation. Photoreceptors subsequently transmit these signals to downstream light-responsive factors, including COP1 and HY5, which regulate auxin pathways and thereby influence *de novo* root organogenesis. The addition of NPA under light enhanced AR regeneration, suggesting that light inhibits AR formation by modulating auxin transport (Blair Nameth et al., 2018). In particular, the efflux of auxin inhibited AR regeneration. Furthermore, the expression of *YUC5/8/9*, enzymes involved in auxin synthesis, was higher in leaf explants in darkness compared to a 16/8-h photoperiod (Chen et al., 2016b). A reduction in the expression of *ARF4/7*, which acted upstream of *LBD16* and promoted AR formation in peach, was also observed during *de novo* root organogenesis in grapevine petioles in response to light exposure (Yuan et al., 2024). These findings suggest that light inhibits *de novo* root organogenesis by regulating auxin synthesis, transport, and signaling. The specific roles of these key factors require further investigation.

Light has also been shown to inhibit the formation of HAR (**Figure 5**). Under light, the active form of PHYB-Pfr interacted with PIFs in the cell nucleus, leading to the phosphorylation of PIF proteins (Bauer et al., 2004) (**Figure 5A**). This interaction inhibited the promotion of HAR by PIFs (Li et al., 2022b). The mechanisms underlying HAR and LR formation were similar. In the case of LR, *IAA14* and *ARF7/19* played key roles. ARF7/19 positively regulated LR formation, whereas IAA14 inhibited this process by suppressing the expression of *ARF7/19* (Goh et al., 2012). PHYB stabilized IAA14 through protein interactions, thereby inhibiting HAR formation by decreasing the transcriptional activity of *ARF7/19* (Li et al., 2021). Additionally, blue light receptors, including CRY1/2, PHOT1/2, and NON-PHOTOTROPIC HYPOCOTYL 3 (NPH3), were involved in regulating HAR under light. NPH3 likely further regulated HAR formation in *Arabidopsis* by modulating auxin transport through the PIN3 protein (Zeng et al., 2022; Zhai et al., 2021). Under dark, PHYB-Pr existed in the cell cytoplasm, and the localization of PIFs in the nucleus directly regulated auxin synthesis and transport (**Figure 5B**). PIFs bound to the promoters of the auxin transporters *AUXIN 1* (*AUX1*) and *LIKE-AUX 3* (*LAX3*), thereby promoting the inward flow of auxin and enhancing HAR regeneration. Furthermore, PIFs directly regulated key transcription factors such as *LBD16/29* and *WOX5/7*, which were involved in the direct promotion of HAR formation (Li et al., 2022b). PIFs are also bound to the promoters of *YUC2/6*, genes involved in auxin synthesis, to increase auxin production, thereby further promoting HAR formation. However, PIFs did not regulate the *YUC5/8/9*, which were highly expressed in the dark, suggesting the involvement of other light signaling factors that mediate auxin synthesis through activation of *YUC5/8/9*. Auxin may also indirectly regulate HAR formation by modulating JA signaling. Three auxin early response genes, *GRETCHEN HAGEN 3.3* (*GH3.3*), *GH3.5*, and *GH3.6*, were positively and redundantly involved in HAR regeneration (Gutierrez et al., 2012). Three proteins interacted with each other (Sorin et al., 2006). The active form of jasmonic acid, jasmonic acid isoleucine (JA-Ile), inhibited HAR formation via the *CORONATINE-INSENSITIVE 1* (*COI1*) signaling pathway (Gutierrez et al., 2012). GH3.3, GH3.5, and GH3.6 reduced JA-Ile levels by promoting the binding of JAs to amino acids such as JA-Met and JA-Asp. *ARF6/8/17*, located upstream of *GH3.3*, *GH3.5*, and *GH3.6*, regulated their expression, with *ARF6/8* positively and *ARF17* negatively regulating *GH3* gene expression (Gutierrez et al., 2012). Auxin upregulated the expression of *GH3.3*, *GH3.5*, and *GH3.6* by activating *ARF6/8*, thereby promoting HAR regeneration through the degradation of JA (Gutierrez et al., 2012). Moreover, ARGONAUTE 1 (AGO1) suppressed the expression of *ARF17* (Sorin et al., 2005).

## 5 Conclusion and future perspectives

Plant cell totipotency is considered one of the 25 most important scientific challenges (Kennedy, 2005), as it enables plant species to undergo tissue repair and organ regeneration in response to injury or stress. In addition to its critical role in maintaining normal physiological functions, plant regeneration forms the basis for the asexual propagation of superior varieties and underpins various biotechnological applications, including genetic transformation and CRISPR-Cas9 (Valencia-Lozano et al., 2024; Deltcheva et al.,2011). Therefore, understanding the factors that influence plant regeneration is essential. Key factors such as the type of explants, culture medium, plant growth regulators, and light conditions all impact the efficiency of plant regeneration (Long et al., 2022). Among these, light conditions have been shown to significantly influence regeneration outcomes. By optimizing light conditions, plant regeneration efficiency can be enhanced. This paper reviews the effects of light intensity, light spectra, and photoperiod on *de novo* shoot organogenesis, somatic embryogenesis, AR formation, and related molecular mechanisms and regulatory networks. These insights contribute to a deeper understanding of the role of light in plant regeneration.

Although significant progress has been made in studying the effects of light on plant regeneration, research has mainly focused on a limited number of plant species. Due to species-specific variations, different species or even different genotypes within the same species can exhibit vastly different responses to light signals. The complex regulation of plant regeneration by light may reflect the evolutionary adaptations of plants, from bryophytes to xerophytes. As a result, the mechanisms underlying light-induced plant regeneration have become increasingly diverse, enabling plants to successfully regenerate even in complex environments. To gain a more comprehensive understanding of how light influences plant regeneration, it is essential to explore the responses of various plant species to light signals. This approach can provide valuable insights and specific references for studying light-regulated regeneration in non-model plants.

Understanding the molecular mechanisms through which light influences plant regeneration is crucial for optimizing the application of light signaling. Currently, most studies on light-induced plant regeneration focus on the model plant *Arabidopsis*. Based on the extensive use of *Arabidopsis* mutants and in-depth studies of light signaling factors, the key light signaling factors involved in regulating plant regeneration have been largely identified. However, through which target genes or transcription factors do light-responsive factors regulate plant regeneration? Which regulatory pathways are involved, and do they interact with each other? Are post-transcriptional regulation and epigenetic modifications involved in light-regulated plant regeneration? The molecular mechanisms of light-regulated regeneration in *Arabidopsis* may be conserved in other species, but differences likely exist. These questions warrant further investigation. Additionally, the mechanisms by which light regulates regeneration in woody plants may differ from those in the herbaceous model *Arabidopsis*. Therefore, it is also crucial to explore the regulatory mechanisms of light in the regeneration of woody plants.

With the rapid advancement of biotechnology, techniques such as CRISPR-Cas9, single-cell multi-omics, spatial genomics, and other technologies have been increasingly applied in plant research. CRISPR-Cas9, in particular, allows for precise gene knockout, insertion, mutation, and modification, making it a powerful tool for improving plant traits and investigating the roles of key factors in light-regulated plant regeneration. Plant regeneration is a complex biological process involving cell and tissue fate transitions, as well as regulation at multiple genetic levels. The emergence of single-cell genomics, genomic data of individual cells, delineation of cellular taxa based on clustering of cells, proposed temporal analysis, and cell trajectory analysis allows us to recognize cell fate transitions during plant regeneration at the cellular level and to gain a deeper understanding of the process of plant regeneration. Additionally, spatial histology, based on tissue sectioning, allows for the observation of individual cell positions and functional states within tissues at a spatial level. For instance, in the regeneration of *de novo* shoot organogenesis, tissue-level changes, as well as associated mRNA and protein alterations throughout the stages—from explant to callus formation to adventitious shoot development—can be captured. These emerging technologies hold great potential for enhancing our understanding of how light influences plant regeneration and will likely play a pivotal role in future research in this area.

## References

[B1] Aalifar M. Arab M. Aliniaeifard S. Dianati S. Zare Mehrjerdi M. Limpens E.. (2019). Embryogenesis efficiency and genetic stability of *Dianthus caryophyllus* embryos in response to different light spectra and plant growth regulators. Plant Cell Tiss Organ Cult. 139, 479–492. doi: 10.1007/s11240-019-01684-6

[B2] Adil M. Ren X. Jeong B. R. (2019). Light elicited growth, antioxidant enzymes activities and production of medicinal compounds in callus culture of *Cnidium officinale* Makino. J. Photochem. Photobiol. B. 196, 111509. doi: 10.1016/j.jphotobiol.2019.05.006 31128431

[B3] Agulló-Antón M.Á. Sánchez-Bravo J. Acosta M. Druege U. (2011). Auxins or Sugars: what makes the difference in the adventitious rooting of stored carnation cuttings? J. Plant Growth Regul. 30, 100–113. doi: 10.1007/s00344-010-9174-8

[B4] Alallaq S. Ranjan A. Brunoni F. Novák O. Lakehal A. Bellini C. (2020). Red light controls adventitious root regeneration by modulating hormone homeostasis in *Picea abies* Seedlings. Front. Plant Sci. 11. doi: 10.3389/fpls.2020.586140 PMC7509059 33014006

[B5] Almeida F. A. Vale E. M. Reis R. S. Santa-Catarina C. Silveira V. (2019). LED lamps enhance somatic embryo maturation in association with the differential accumulation of proteins in the *Carica papaya* L. ‘Golden’ embryogenic callus. Plant Physiol. Bioch. 143, 109–118. doi: 10.1016/j.plaphy.2019.08.029 31491701

[B6] Assou J. Bethge H. Wamhoff D. Winkelmann T. (2023). Effect of cytokinins and light quality on adventitious shoot regeneration from leaflet explants of peanut (*Arachis hypogaea*). J. Hortic. Sci. Biotech. 98, 508–525. doi: 10.1080/14620316.2022.2160382

[B7] Atta R. Laurens L. Boucheron-Dubuisson E. Guivarc’h A. Carnero E. Giraudat-Pautot V.. (2009). Pluripotency of *Arabidopsis* xylem pericycle underlies shoot regeneration from root and hypocotyl explants grown in *vitro* . Plant J. 57, 626–644. doi: 10.1111/j.1365-313X.2008.03715.x 18980654

[B8] Bakhshaie M. Babalar M. Mirmasoumi M. Khalighi A. (2010). Effects of light, sucrose, and cytokinins on somatic embryogenesis in *Lilium ledebourii* (Baker) Bioss. via transverse thin cell-layer cultures of bulblet microscales. J. Hortic. Sci. Biotech. 85, 491–496. doi: 10.1080/14620316.2010.11512703

[B9] Baque M. Hahn E.-J. Paek K.-Y. (2010). Induction mechanism of adventitious root from leaf explants of *Morinda citrifolia* as affected by auxin and light quality. In Vitro Cell.Dev.Biol.-Plant 46, 71–80. doi: 10.1007/s11627-009-9261-3

[B10] Bauer D. Viczián A. Kircher S. Nobis T. Nitschke R. Kunkel T.. (2004). Constitutive photomorphogenesis 1 and multiple photoreceptors control degradation of phytochrome interacting factor 3, a transcription factor required for light signaling in *Arabidopsis* . Plant Cell. 16, 1433–1445. doi: 10.1105/tpc.021568 15155879 PMC490037

[B11] Belić M. Zdravković-Korać S. Janošević D. Savić J. Todorović S. Banjac N.. (2020). Gibberellins and light synergistically promote somatic embryogenesis from the *in vitro* apical root sections of spinach. Plant Cell Tiss Organ Cult. 142, 537–548. doi: 10.1007/s11240-020-01878-3

[B12] Berckmans B. Vassileva V. Schmid S. P. C. Maes S. Parizot B. Naramoto S.. (2011). Auxin-dependent cell cycle reactivation through transcriptional regulation of *Arabidopsis* E2Fa by lateral organ boundary proteins. Plant Cell. 23, 3671–3683. doi: 10.1105/tpc.111.088377 22003076 PMC3229142

[B13] Bhatnagar A. Singh S. Khurana J. P. Burman N. (2020). HY5-COP1: the central module of light signaling pathway. J. Plant Biochem. Biotechnol. 29, 590–610. doi: 10.1007/s13562-020-00623-3

[B14] Biswal B. Jena B. Giri A. K. Acharya L. (2022). Monochromatic light elicited biomass accumulation, antioxidant activity, and secondary metabolite production in callus culture of *Operculina turpethum* (L.). Plant Cell Tiss Organ Cult. 149, 123–134. doi: 10.1007/s11240-022-02274-9

[B15] Biswas M. K. Islam R. Hossain M. (2007). Somatic embryogenesis in strawberry (*Fragaria* sp.) through callus culture. Plant Cell Tiss Organ Cult. 90, 49–54. doi: 10.1007/s11240-007-9247-y

[B16] Blair Nameth M. Goron T. L. Dinka S. J. Morris A. D. English J. Lewis D.. (2018). The initial hours of post-excision light are critical for adventitious root regeneration from *Arabidopsis thaliana* (L.) Heynh. cotyledon explants. In Vitro Cell. Dev. Biol.-Plant 54, 273–290. doi: 10.1007/s11627-017-9880-z

[B17] Brand U. Grünewald M. Hobe M. Simon R. (2002). Regulation of CLV3 expression by two homeobox genes in *Arabidopsis* . Plant Physiol. 129, 565–575. doi: 10.1104/pp.001867 12068101 PMC161677

[B18] Braybrook S. A. Stone S. L. Park S. Bui A. Q. Le B. H. Fischer R. L.. (2006). Genes directly regulated by LEAFY COTYLEDON 2 provide insight in-to the control of embryo maturation and somatic embryogenesis. Proc. Natl. Acad. Sci. U.S.A. 103, 3468–3473. doi: 10.1073/pnas.0511331103 16492731 PMC1413938

[B19] Buechel S. Leibfried A. To J. P. C. Zhao Z. Andersen S. U. Kieber J. J.. (2010). Role of A-type ARABIDOPSIS RESPONSE REGULATORS in meri-stem maintenance and regeneration. Eur. J. Cell Biol. 89, 279–284. doi: 10.1016/j.ejcb.2009.11.016 20018401

[B20] Burritt D. J. Leung D. W. M. (2003). Adventitious shoot regeneration from *Begonia × erythrophylla* petiole sections is developmentally sensitive to light quality. Physiologia Plantarum. 118, 289–296. doi: 10.1034/j.1399-3054.2003.00083.x

[B21] Caboni E. Lauri P. D’Angeli S. (2000). *In vitro* plant regeneration from callus of shoot apices in apple shoot culture. Plant Cell Rep. 19, 755–760. doi: 10.1007/s002999900189 30754865

[B22] Caillot S. Rosiau E. Laplace C. Thomasset B. (2009). Influence of light intensity and selection scheme on regeneration time of transgenic flax plants. Plant Cell Rep. 28, 359–371. doi: 10.1007/s00299-008-0638-2 19011860

[B23] Castander-Olarieta A. Montalbán I. A. Moncaleán P. (2023). Multi-strategy ap-proach towards optimization of maturation and germination in radiata pine so-matic embryogenesis. Plant Cell Tiss Organ Cult. 153, 173–190. doi: 10.1007/s11240-023-02457-y

[B24] Chan A. Stasolla C. (2023). Light induction of somatic embryogenesis in *Arabidopsis* is regulated by PHYTOCHROME E. Plant Physiol. Biotech. 195, 163–169. doi: 10.1016/j.plaphy.2023.01.007 36640683

[B25] Chen C.-C. Agrawal D. C. Lee M.-R. Lee R.-J. Kuo C.-L. Wu C.-R.. (2016a). Influence of LED light spectra on *in vitro* somatic embryogenesis and LC–MS analysis of chloro-genic acid and rutin in *Peucedanum japonicum* Thunb.: a medicinal herb. Bot. Stud. 57, 9. doi: 10.1186/s40529-016-0124-z 28597418 PMC5430566

[B26] Chen J.-R. Wu L. Hu B.-W. Yi X. Liu R. Deng Z.-N.. (2014). The Influence of plant growth regulators and light quality on somatic embryogenesis in China Rose (*Rosa chinensis* Jacq.). J. Plant Growth Regul. 33, 295–304. doi: 10.1007/s00344-013-9371-3

[B27] Chen L. Tong J. Xiao L. Ruan Y. Liu J. Zeng M.. (2016b). YUCCA -mediated auxin biogenesis is required for cell fate transition occurring during *de novo* root organogenesis in *Arabidopsis* . J. Exp. Bot. 67, 4273–4284. doi: 10.1093/jxb/erw213 27255928 PMC5301932

[B28] Chen Y. Hu Y. Wang R. Feng K. Di J. Feng T.. (2023). Transcriptome and physiological analysis highlight the hormone, phenylpropanoid, and photosynthesis effects on early somatic embryogenesis in *Ginkgo biloba* . Ind. Crop Prod. 203, 117176. doi: 10.1016/j.indcrop.2023.117176

[B29] Chen Y.-M. Huang J.-Z. Hou T.-W. Pan I.-C. (2019). Effects of light intensity and plant growth regulators on callus proliferation and shoot regeneration in the ornamental succulent *Haworthia* . Bot. Stud. 60, 10. doi: 10.1186/s40529-019-0257-y 31267253 PMC6606681

[B30] Cheng L. Han Y. Zhao M. Xu X. Shen Z. Wang C.. (2020). Gene expression profiling reveals the effects of light on adventitious root formation in lotus seedlings (*Nelumbo nucifera* Gaertn.). BMC Genom. 21 (1), 707. doi: 10.1186/s12864-020-07098-5 PMC7552355 33045982

[B31] Cheng M. C. Kathare P. K. Paik I. Huq E. (2021a). Phytochrome signaling networks. Annu. Rev. Plant Biol. 72, 217–244. doi: 10.1146/annurev-arplant-080620-024221 33756095 PMC10988782

[B32] Cheng Y. Liu H. Tong X. Liu Z. Zhang X. Jiang X.. (2021b). Somatic embryo-genesis and triterpenoid saponin production in *Aralia elata* (Miq.) Seem. Sci. Hortic. 285, 110162. doi: 10.1016/j.scienta.2021.110162

[B33] Christiaens A. Gobin B. Van Huylenbroeck J. Van Labeke M.-C. (2019). Adventitious rooting of *Chrysanthemum* is stimulated by a low red: far-red ratio. J. Plant Physiol. 236, 117–123. doi: 10.1016/j.jplph.2019.03.008 30974405

[B34] Christie J. M. (2007). Phototropin blue-light receptors. Annu. Rev. Plant Biol. 58, 21–45. doi: 10.1146/annurev.arplant.58.032806.103951 17067285

[B35] Dai X. Wang J. Song Y. Liu Z. Xue T. Qiao M.. (2021). Cytosine methylation of the FWA promoter promotes direct *in vitro* shoot regeneration in *Arabidopsis thaliana* . J. Integr. Plant Biol. 63, 1491–1504. doi: 10.1111/jipb.13156 34292662

[B36] Dai X. Wang J. Wang L. Liu Z. Li Q. Cai Y.. (2022). HY5 inhibits in vitro shoot stem cell niches initiation via directly repressing pluripotency and cytokinin pathways. Plant J. 110, 781–801. doi: 10.1111/tpj.15703 35132706

[B37] Deltcheva E. Chylinski K. Sharma C. M. Gonzales K. Chao Y. Pirzada Z. A.. (2011). CRISPR RNA maturation by trans-encoded small RNA and host fac-tor RNase III. Nature 471, 602–607. doi: 10.1038/nature09886 21455174 PMC3070239

[B38] Dobránszki J. Teixeira Da Silva J. A. (2011). Adventitious shoot regeneration from leaf thin cell layers in apple. Sci. Hortic. 127, 460–463. doi: 10.1016/j.scienta.2010.11.003

[B39] Dong N. Montanez B. Creelman R. A. Cornish K. (2006). Low light and low ammonium are key factors for guayule leaf tissue shoot organogenesis and transformation. Plant Cell Rep. 25, 26–34. doi: 10.1007/s00299-005-0024-2 16247613

[B40] Duclercq J. Sangwan-Norreel B. Catterou M. Sangwan R. S. (2011). *De novo* shoot organogenesis: from art to science. Trends Plant Sci. 16, 597–606. doi: 10.1016/j.tplants.2011.08.004 21907610

[B41] Dutta Gupta S. Karmakar A. (2017). Machine vision based evaluation of impact of light emitting diodes (LEDs) on shoot regeneration and the effect of spectral quality on phenolic content and antioxidant capacity in *Swertia chirata* . J. Photochem. Photobiol. B 174, 162–172. doi: 10.1016/j.jphotobiol.2017.07.029 28779689

[B42] Elhiti M. Hebelstrup K. H. Wang A. Li C. Cui Y. Hill R. D.. (2013). Function of type–2 *Arabidopsis* hemoglobin in the auxin-mediated formation of embryogenic cells during morphogenesis. Plant J. 74, 946–958. doi: 10.1111/tpj.12181 23510449

[B43] Elmeer K. M. S. Hennerty M. J. (2008). Observations on the combined effects of light, NAA and 2,4-D on somatic embryogenesis of cucumber (*Cucumis sativus*) hybrids. Plant Cell Tiss Organ Cult. 95, 381–384. doi: 10.1007/s11240-008-9439-0

[B44] Espinosa A. C. Pijut P. M. Michler C. H. (2006). Adventitious shoot regeneration and rooting of *Prunus serotina In Vitro* Cultures. HortSci. 41, 193–201. doi: 10.21273/HORTSCI.41.1.193

[B45] Fan M. Xu C. Xu K. Hu Y. (2012). LATERAL ORGAN BOUNDARIES DO-MAIN transcription factors direct callus formation in *Arabidopsis* regeneration. Cell Res. 22, 1169–1180. doi: 10.1038/cr.2012.63 22508267 PMC3391013

[B46] Farhadi N. Panahandeh J. Azar A. M. Salte S. A. (2017). Effects of explant type, growth regulators and light intensity on callus induction and plant regeneration in four ecotypes of Persian shallot (*Allium hirtifolium*). Sci. Hortic-amsterdam. 218, 80–86. doi: 10.1016/j.scienta.2016.11.056

[B47] Fehér A. (2015). Somatic embryogenesis - Stress-induced remodeling of plant cell fate. Biochim. Biophys. Acta 1849, 385–402. doi: 10.1016/j.bbagrm.2014.07.005 25038583

[B48] Feldman L. J. (1976). The *de novo* origin of the quiescent center regenerating root apices of *Zea mays* . Planta 128, 207–212. doi: 10.1007/BF00393230 24430748

[B49] Fett-Neto A. G. Fett J. P. Goulart L. W. V. Pasquali G. Termignoni R. R. Ferreira A. G. (2001). Distinct effects of auxin and light on adventitious root development in *Eucalyptus saligna* and *Eucalyptus globulu*s. Tree Physiol. 21, 457–464. doi: 10.1093/treephys/21.7.457 11340046

[B50] Fletcher R. A. Peterson R. L. Zalik S. (1965). Effect of light quality on elongation, adventitious root production and the relation of cell number and cell size to bean seedling elongation. Plant Physiol. 40, 541–548. doi: 10.1104/pp.40.3.541 16656122 PMC550329

[B51] Franklin K. A. Quail P. H. (2010). Phytochrome functions in *Arabidopsis* development. J. Exp. Bot. 61, 11–24. doi: 10.1093/jxb/erp304 19815685 PMC2800801

[B52] Fu H. Wei X. Chen Q. Yong S. Liu Q. Dang J.. (2023). Comparative transcriptome analysis of molecular mechanisms underlying adventitious root developments in Huangshan Bitter tea (*Camellia gymnogyna Chang*) under red light quality. Front. Plant Sci. 14. doi: 10.3389/fpls.2023.1154169 PMC10070859 37025148

[B53] Fuernkranz H. A. Nowak C. A. Maynard C. A. (1990). Light effects on *in vitro* adventitious root formation in axillary shoots of mature *Prunus serotina* . Physiologia Plantarum. 80, 337–341. doi: 10.1111/j.13993054.1990.tb00050.x

[B54] Gao Y. Cui Y. Zhao R. Chen X. Zhang J. Zhao J.. (2022). Cryo-treatment enhances the embryogenicity of mature somatic embryos via the lncRNA–miRNA–mRNA network in white spruce. Int. J. Mol. Sci. 23, 1111. doi: 10.3390/ijms23031111 35163033 PMC8834816

[B55] Gao F. Peng C. Wang H. Tretyakova I. N. Nosov A. M. Shen H.. (2020). Key techniques for somatic embryogenesis and plant regeneration of *Pinus koraiensis* . Forests 11, 912. doi: 10.3390/f11090912

[B56] Gentile A. Monticelli S. Damiano C. (2002). Adventitious shoot regeneration in peach [*Prunus persica* (L.) Batsch. Plant Cell Rep. 20, 1011–1016. doi: 10.1007/s00299-002-0451-2

[B57] Ghadirzadeh-Khorzoghi E. Jahanbakhshian-Davaran Z. Seyedi S. M. (2019). Direct somatic embryogenesis of drought resistance pistachio (*Pistacia vera* L.) and expression analysis of somatic embryogenesis-related genes. S. Afr. J. Bot. 121, 558–567. doi: 10.1016/j.sajb.2019.01.023

[B58] Gil C. S. Jung H. Y. Lee C. Eom S. H. (2020). Blue light and NAA treatment significantly improve rooting on single leaf-bud cutting of *Chrysanthemum* via upregulated rooting-related genes. Scientia Horticulturae. 274, 109650. doi: 10.1016/j.scienta.2020.109650

[B59] Goh T. Kasahara H. Mimura T. Kamiya Y. Fukaki H. (2012). Multiple AUX/IAA-ARF modules regulate lateral root formation: the role of Arabidopsis SHY2/IAA3-mediated auxin signalling. Philos Trans R Soc Lond B Biol Sci. 367 (1595), 1461–8. doi: 10.1098/rstb.2011.0232 22527388 PMC3321683

[B60] Gutierrez L. Bussell J. D. Păcurar D. I. Schwambach J. Păcurar M. Bellini C. (2009). Phenotypic plasticity of adventitious rooting in *Arabidopsis* is con-trolled by complex regulation of auxin response factor transcripts and microRNA abundance. Plant Cell. 21, 3119–3132. doi: 10.1105/tpc.108.064758 19820192 PMC2782293

[B61] Gutierrez L. Mongelard G. Floková K. Păcurar D. I. Novák O. Staswick P.. (2012). Auxin controls *Arabidopsis* adventitious root initiation by regulating jasmonic acid homeostasis. Plant Cell. 24, 2515–2527. doi: 10.1105/tpc.112.099119 22730403 PMC3406919

[B62] Halperin W. (1966). Alternative morphogenetic events in cell suspensions. Am. J. Bot. 53, 443–453. doi: 10.1002/j.1537-2197.1966.tb07357.x

[B63] Hansen J. (1975). Light dependent promotion and inhibition of adventitious root formation by gibberellic acid. Planta 123, 203–205. doi: 10.1007/BF00383871 24435089

[B64] Hassanpour H. (2022). Potential impact of red-blue LED light on callus growth, cell viability, and secondary metabolism of *Hyoscyamus reticulatus* . In Vitro Cell. Dev. Biol.-Plant 58, 256–265. doi: 10.1007/s11627-021-10232-x

[B65] Heringer A. S. Reis R. S. Passamani L. Z. De Souza-Filho G. A. Santa-Catarina C. Silveira V. (2017). Comparative proteomics analysis of the effect of combined red and blue lights on sugarcane somatic embryogenesis. Acta Physiol. Plant 39, 52. doi: 10.1007/s11738-017-2349-1

[B66] Hernández-Piedra G. Ruiz-Carrera V. Sánchez A. J. Hernández-Franyutti A. Azpeitia-Morales A. (2018). Morpho-histological development of the somatic embryos of *Typha domingensis* . PeerJ 6, e5952. doi: 10.7717/peerj.5952 30505633 PMC6254243

[B67] Hesami M. Naderi R. Tohidfar M. Yoosefzadeh-Najafabadi M. (2019). Application of adaptive neuro-fuzzy inference system-non-dominated sorting genetic algorithm-II (AN-FIS-NSGAII) for modeling and optimizing somatic embryogenesis of *Chrysanthemum* . Front. Plant Sci. 10. doi: 10.3389/fpls.2019.00869 PMC6624437 31333705

[B68] Horstman A. Li M. Heidmann I. Weemen M. Chen B. Muino J. M.. (2017). The BABY BOOM transcription factor activates the LEC1-ABI3-FUS3-LEC2 network to induce somatic embryogenesis. Plant Physiol. 175, 848–857. doi: 10.1104/pp.17.00232 28830937 PMC5619889

[B69] Hu X. Xu L. (2016). Transcription factors WOX11/12 directly activate WOX5/7 to promote root primordia initiation and organogenesis. Plant Physiol. 172, 2363–2373. doi: 10.1104/pp.16.01067 27784768 PMC5129711

[B70] Hua W. Zhu J Shang Y Wang J Jia Q Lin F (2013). Establishment of a highly efficient regeneration method from the scraped broken embryo of mature barley seed. Can J Plant Sci. 93 (6), 1029–1035. doi: 10.4141/cjps2013-109

[B71] Iacona C. Muleo R. (2010). Light quality affects *in vitro* adventitious rooting and ex vitro performance of cherry rootstock Colt. Sci. Hortic. 125, 630–636. doi: 10.1016/j.scienta.2010.05.018

[B72] Ikeda-Iwai M. Umehara M. Satoh S. Kamada H. (2003). Stress-induced somatic embryogenesis in vegetative tissues of *Arabidopsis thaliana* . Plant J. 34, 107–114. doi: 10.1046/j.1365-313X.2003.01702.x 12662313

[B73] Jeong B. R. Sivanesan I. (2018). Impact of light quality and sucrose on adventitious shoot regeneration and bioactive compound accumulation in *Ajuga multiflora Bunge* . Sci. Hortic. 236, 222–228. doi: 10.1016/j.scienta.2018.03.056

[B74] Jing D. Zhang J. Xia Y. Kong L. OuYang F. Zhang S.. (2017). Proteomic analysis of stress-related proteins and metabolic pathways in *Picea asperata* so-matic embryos during partial desiccation. Plant Biotechnol. J. 15, 27–38. doi: 10.1111/pbi.12588 27271942 PMC5253475

[B75] Kami C. Lorrain S. Hornitschek P. Fankhauser C. (2010). Light-regulated plant growth and development. Curr. Top. Dev. Biol. 91, 29–66. doi: 10.1016/S0070-2153(10)91002-8 20705178

[B76] Kapoor S. Raghuvanshi R. Bhardwaj P. Sood H. Saxena S. Chaurasia O. P. (2018). Influence of light quality on growth, secondary metabolites production and antioxidant activity in callus culture of *Rhodiola imbricata* Edgew. J. Photochem. Photobiol. B. 183, 258–265. doi: 10.1016/j.jphotobiol.2018.04.018 29747145

[B77] Karami O. Philipsen C. Rahimi A. Nurillah A. R. Boutilier K. Offringa R. (2023). Endogenous auxin maintains embryonic cell identity and promotes somatic embryo development in *Arabidopsis* . Plant J. 113, 7–22. doi: 10.1111/tpj.16024 36345646 PMC10098609

[B78] Kareem A. Durgaprasad K. Sugimoto K. Du Y. Pulianmackal AJ. Trivedi Z (2015). PLETHORA Genes Control Regeneration by a Two-Step Mechanism. Curr Biol. 25 (8), 1017–30. doi: 10.1016/j.cub.2015.02.022 25819565 PMC4829346

[B79] Kareem A. Radhakrishnan D. Sondhi Y. Aiyaz M. Roy M. Sugimoto K. (2016). De novo assembly of plant body plan: a step ahead of Deadpool. Regeneration (Oxf). 3 (4), 182–197. doi: 10.1002/reg2.68 27800169 PMC5084358

[B80] Kennedy D. (2005). 125th anniversary series. Science 309, 19–19. doi: 10.1126/science.1115951 15994489

[B81] Khatri P. Joshee N. (2024). Effect of picloram and desiccation on the somatic embryogenesis of *Lycium barbarum* L. Plants 13, 151. doi: 10.3390/plants13020151 38256705 PMC10820025

[B82] Klopotek Y. Haensch K.-T. Hause B. Hajirezaei M.-R. Druege U. (2010). Dark exposure of petunia cuttings strongly improves adventitious root formation and enhances carbohydrate availability during rooting in the light. J. Plant Physiol. 167, 547–554. doi: 10.1016/j.jplph.2009.11.008 20047776

[B83] Krzemińska M. Hnatuszko-Konka K. Weremczuk-Jeżyna I. Owczarek-Januszkiewicz A. Ejsmont W. Olszewska M. A.. (2023). Effect of light conditions on polyphenol production in transformed shoot culture of *Salvia bulleyana* Diels. Molecules 28, 4603. doi: 10.3390/molecules28124603 37375158 PMC10302416

[B84] Kumar A. Kumar V. A. Kumar J. (1993). Rapid *in vitro* propagation of cauliflower. Plant Science. 90, 175–178. doi: 10.1016/0168-9452(93)90237-T

[B85] Kwaaitaal M. A. C. J. De Vries S. C. (2007). The SERK1 gene is expressed in pro-cambium and immature vascular cells. J. Exp. Bot. 58, 2887–2896. doi: 10.1093/jxb/erm103 17630293

[B86] Kwon A. R. Cui H. Y. Lee H. Shin H. Kang K. S. Park S. Y. (2015). Light quality affects shoot regeneration, cell division, and wood formation in elite clones of Populus euramericana. Acta Physiol Plant 37, 65–1514. doi: 10.1007/s11738-015-1812-0

[B87] Lakehal A. Chaabouni S. Cavel E. Le Hir R. Ranjan A. Raneshan Z.. (2019). A molecular framework for the control of adventitious rooting by TIR1/AFB2-Aux/IAA-dependent suxin signaling in *Arabidopsis* . Mol. Plant 12, 1499–1514. doi: 10.1016/j.molp.2019.09.001 31520787

[B88] Lee H. W. Cho C. Pandey S. K. Park Y. Kim M.-J. Kim J. (2019). LBD16 and LBD18 acting downstream of ARF7 and ARF19 are involved in adventitious root formation in *Arabidopsis* . BMC Plant Biol. 19, 46. doi: 10.1186/s12870-019-1659-4 30704405 PMC6357364

[B89] Lee H. W. Kim M. Kim N. Y. Lee S. H. Kim J. (2013). LBD18 acts as a tran-scriptional activator that directly binds to the EXPANSIN14 promoter in promoting lateral root emergence of *Arabidopsis* . Plant J. 73, 212–224. doi: 10.1111/tpj.12013 22974309

[B90] Lercari B. Bertram L. (2004). Interactions of phytochromes A, B1 and B2 in light-induced competence for adventitious shoot formation in hypocotyl of tomato (*Solanum lycopersicum* L.). Plant Cell Rep. 22, 523–531. doi: 10.1007/s00299-003-0725-3 14600782

[B91] Leshem B. Ronen R. Soudry E. Lurie S. Gepstein S. (1995). Cytokinin and white light coact to enhance polypeptide metabolism and shoot regeneration in cultured melon cotyledons. J. Plant Physiol. 145, 291–295. doi: 10.1016/S0176-1617(11)81892-4

[B92] Li M. Wrobel-Marek J. Heidmann I. Horstman A. Chen B. Reis R.. (2022a). Auxin biosynthesis maintains embryo identity and growth during BABY BOOM-induced somatic embryogenesis. Plant Physiol. 188, 1095–1110. doi: 10.1093/plphys/kiab558 34865162 PMC8825264

[B93] Li Q. Zhang Z. Wang Y. Zhong L. Chao Z. Gao Y.. (2021). Phytochrome B inhibits darkness-induced hypocotyl adventitious root formation by stabilizing IAA14 and suppressing ARF7 and ARF19. Plant J. 105, 1689–1702. doi: 10.1111/tpj.15142 33354819

[B94] Li Q.-Q. Zhang Z. Zhang C.-X. Wang Y.-L. Liu C.-B. Wu J.-C.. (2022b). Phytochrome-interacting factors orchestrate hypocotyl adventitious root initiation in *Arabidopsis* . Development 149, dev200362. doi: 10.1242/dev.200362 35502748

[B95] Libao C. Yuyan H. Minrong Z. Xiaoyong X. Zhiguang S. Chunfei W.. (2020). Gene expression profiling reveals the effects of light on adventitious root formation in lotus seedlings (*Nelumbo nucifera* Gaertn.). BMC Genom. 21 (1), 707. doi: 10.1186/s12864-020-07098-5 PMC7552355 33045982

[B96] Lim M.-J. Murthy H. N. Song H.-Y. Lee S.-Y. Park S.-Y. (2023). Influence of white, red, blue, and combination of LED lights on *in vitro* multiplication of shoots, rooting, and acclimatization of *Gerbera jamesonii* cv. ‘Shy Pink’ Plants. Agronomy 13, 2216. doi: 10.3390/agronomy13092216

[B97] Lin C. S. Hsu C. T. Yang L. H. Lee L. Y. Fu J. Y. Cheng Q. W.. (2018). Application of protoplast technology to CRISPR/Cas9 mutagenesis: from single-cell mutation detection to mutant plant regeneration. Plant Biotechnol. J. 16, 1295–1310. doi: 10.1111/pbi.12870 29230929 PMC5999315

[B98] Liu J. H. Dong W. C. Fei F. F. Li X. T. Zhang X. H. Zhou Y.. (2022). Regulation of WOX11 expression represents the difference between direct and indirect shoot regeneration. Front. Plant Sci. 13. doi: 10.3389/fpls.2022.850726 PMC8931721 35310629

[B99] Liu W. Zhang Y. Fang X. Tran S. Zhai N. Yang Z.. (2022). Transcriptional landscapes of *de novo* root regeneration from detached *Arabidopsis* leaves revealed by time-lapse and single-cell RNA sequencing analyses. Plant Commun. 3, 100306. doi: 10.1016/j.xplc.2022.100306 35605192 PMC9284295

[B100] Liu C. Moon K. Honda H. Kobayashi D. T. (2001). Enhanced regeneration of rice (*Oryza sativa* L.) embryogenic callus by light irradiation in growth phase. J. Biosci. Bioeng. 91, 319–321. doi: 10.1016/S1389-1723(01)80143-2 16232998

[B101] Liu J. Sheng L. Xu Y. Li J. Yang Z. Huang H.. (2014). WOX11 and 12 are involved in the first-step cell fate transition during *de Novo* root organogenesis in *Arabidopsis* . Plant Cell. 26, 1081–1093. doi: 10.1105/tpc.114.122887 24642937 PMC4001370

[B102] Long Y. Yang Y. Pan G. Shen Y. (2022). New insights into tissue culture plant-regeneration mechanisms. Front. Plant Sci. 13. doi: 10.3389/fpls.2022.926752 PMC9280033 35845646

[B103] Loshyna L. Bulko О. Kuchuk M. (2022). Adventitious regeneration of blackberry and raspberry shoots and the assessment of the LED-lighting impact. Zemdirbyste-Agriculture 109, 49–54. doi: 10.13080/z-a.2022.109.007

[B104] Lu X.-D. Zhou C.-M. Xu P.-B. Luo Q. Lian H.-L. Yang H.-Q. (2015). Red-light-dependent interaction of phyB with SPA1 promotes COP1–SPA1 dissociation and photomorphogenic development in *Arabidopsis* . Mol. Plant 8, 467–478. doi: 10.1016/j.molp.2014.11.025 25744387

[B105] Mazri M. A. Naciri R. Belkoura I. (2020). Maturation and conversion of somatic embryos derived from seeds of olive (*Olea europaea* L.) cv. Dahbia: occurrence of secondary embryogenesis and adventitious bud formation. Plants 9, 1489. doi: 10.3390/plants9111489 33158272 PMC7694239

[B106] Melo N. K. G. Bianchetti R. E. Lira B. S. Oliveira P. M. R. Zuccarelli R. Dias D. L. O.. (2016). nitric oxide, ethylene, and auxin cross talk mediates greening and plastid development in deetiolating tomato seedlings. Plant Physiol. 170, 2278–2294. doi: 10.1104/pp.16.00023 26829981 PMC4825133

[B107] Meziani R. Jaiti F. Mazri M. A. Anjarne M. Chitt M. A. El Fadile J.. (2015). Effects of plant growth regulators and light intensity on the micropropagation of date palm (*Phoenix dactylifera* L.) cv. Mejhoul. J. Crop Sci. Biotechnol. 18, 325–331. doi: 10.1007/s12892-015-0062-4

[B108] Mikuła A. Grzyb M. Rybczyński J. J. (2015). An unique system of somatic embryogenesis in the tree fern *Cyathea delgadii* Sternb.: the importance of explant type, and physical and chemical factors. Plant Cell Tiss Organ Cult. 123, 467–478. doi: 10.1007/s11240-015-0850-z

[B109] Milojević J. Tubić L. Pavlović S. Mitić N. Ćalić D. Vinterhalter B.. (2012). Long days promote somatic embryogenesis in spinach. Sci. Hortic. 142, 32–37. doi: 10.1016/j.scienta.2012.04.020

[B110] Mira M. M. Day S. Ibrahim S. Hill R. D. Stasolla C. (2023). The *Arabidopsis* Phytoglobin 2 mediates phytochrome B (phyB) light signaling responses during somatic embryogenesis. Planta 257, 88. doi: 10.1007/s00425-023-04121-3 36976396

[B111] Miroshnichenko D. N. Filippov M. V. Dolgov S. V. (2013). Medium optimization for efficient somatic embryogenesis and *in vitro* plant regeneration of spring common wheat varieties. Russ. Agricult. Sci. 39, 24–28. doi: 10.3103/S1068367413010175

[B112] Moradi S. Dianati Daylami S. Arab M. Vahdati K. (2017). Direct somatic embryogenesis in *Epipactis veratrifolia*, a temperate terrestrial orchid. J. Hortic. Sci. Biotechnol. 92, 88–97. doi: 10.1080/14620316.2016.1228434

[B113] Naderi Boldaji H. Dianati Daylami S. Vahdati K. (2023). Use of light Spectra for efficient production of PLBs in temperate terrestrial orchids. Horticulturae 9, 1007. doi: 10.3390/horticulturae9091007

[B114] Nameth B. Dinka S. J. Chatfield S. P. Morris A. English J. Lewis D.. (2013). The shoot regeneration capacity of excised *Arabidopsis* cotyledons is established during the initial hours after injury and is modulated by a complex genetic network of light signalling. Plant Cell Environ. 36, 68–86. doi: 10.1111/j.1365-3040.2012.02554.x 22681544

[B115] Nameth M. B. Goron T. L. Dinka S. J. Morris A. D. English J. Lewis D. (2018). The initial hours of post-excision light are critical for adventitious root regeneration from *Arabidopsis thaliana* (L.) Heynh. cotyledon explants. In Vitro Cell. Dev. Biol.-Plant 54, 273–90. doi: 10.1007/s11627-017-9880-z

[B116] Nawrot-Chorabik K. (2016). Plantlet regeneration through somatic embryogenesis in Nord-mann’s fir (*Abies nordmanniana*). J. For. Res. 27, 1219–1228. doi: 10.1007/s11676-016-0265-7

[B117] Negin B. Shemer O. Sorek Y. Eshed Williams L. (2017). Shoot stem cell specification in roots by the *WUSCHEL* transcription factor. PloS One 12, e0176093. doi: 10.1371/journal.pone.0176093 28445492 PMC5405954

[B118] Nhut D. T. Huy N. P. Tai N. T. Nam N. B. Luan V. Q. Hien V. T.. (2015). Light-emitting diodes and their potential in callus growth, plantlet development and saponin ac-cumulation during somatic embryogenesis of *Panax Vietnamensis* Ha et Grushv. Biotechnol. Biotechnol. Equip. 29, 299–308. doi: 10.1080/13102818.2014.1000210 26019644 PMC4433904

[B119] Ouyang J. Wang X. Zhao B. Wang Y. (2003). Light intensity and spectral quality influencing the callus growth of *Cistanche deserticola* and biosynthesis of phenylethanoid glycosides. Plant Sci. 165, 657–661. doi: 10.1016/S0168-9452(03)00255-3

[B120] Pan J. Song J. Sohail H. Sharif R. Yan W. Hu Q.. (2024). RNA-seq-based comparative transcriptome analysis reveals the role of CsPrx73 in waterlogging-triggered adventitious root formation in cucumber. Hortic. Res. 11, uhae062. doi: 10.1093/hr/uhae062 38659441 PMC11040206

[B121] Park S.-Y. Yeung E. C. Paek K.-Y. (2010). Endoreduplication in *Phalaenopsis* is affected by light quality from light-emitting diodes during somatic embryogenesis. Plant Biotechnol. Rep. 4, 303–309. doi: 10.1007/s11816-010-0148-x

[B122] Pashkovskiy P. P. Soshinkova T. N. Korolkova D. V. Kartashov A. V. Zlobin I. E. Lyubi-mov. (2018). The effect of light quality on the pro-/antioxidant balance, activity of photosystem II, and expression of light-dependent genes in *Eutrema salsugineum* callus cells. Photosynth Res. 136, 199–214. doi: 10.1007/s11120-017-0459-7 29071562

[B123] Pinto G. Park Y.-S. Silva S. Neves L. Araújo C. Santos C. (2008). Factors affecting maintenance, proliferation, and germination of secondary somatic embryos of *Eucalyptus globulus* Labill. Plant Cell Tiss Organ Cult. 95, 69–78. doi: 10.1007/s11240-008-9417-6

[B124] Pudasaini A. Zoltowski B. D. (2013). Zeitlupe senses blue-light fluence to mediate circadian timing in *Arabidopsis thaliana* . Biochemistry 52, 7150–7158. doi: 10.1021/bi401027n 24033190

[B125] Raghavan V. (2004). Role of 2,4-dichlorophenoxyacetic acid (2,4-D) in somatic embryogenesis on cultured zygotic embryos of *Arabidopsis*: cell expansion, cell cycling, and morphogenesis during continuous exposure of embryos to 2,4-D. Am. J. Botany. 91, 1743–1756. doi: 10.3732/ajb.91.11.1743 21652321

[B126] Reinert J. (1958). Morphogenesis and its control in tissue cultures from carrots. Sci. Nat. 45, 344–345. doi: 10.1007/BF00640240

[B127] Reuveni M. Evenor D. (2007). On the effect of light on shoot regeneration in petunia. Plant Cell Tiss Organ Cult. 89, 49–54. doi: 10.1007/s11240-007-9215-6

[B128] Rianawati S. Dasumiati D. Rahmi N. A. Kurniati R. Mulyaningsih E. S. (2023). The of effects of light and a combination of growth regulators on the induction of somatic embryogenesis in *Orchid Rhynchostylis gigantea* (LindI.). Ridl. JSM 52, 3091–3102. doi: 10.17576/jsm-2023-5211-06

[B129] Rikiishi K. Matsuura T. Ikeda Y. Maekawa M. (2015). Light inhibition of shoot regeneration is regulated by endogenous abscisic acid level in calli derived from immature barley embryos. PloS One 10, e0145242. doi: 10.1371/journal.pone.0145242 26670930 PMC4682856

[B130] Rikiishi K. Matsuura T. Maekawa M. Takeda K. (2008). Light control of shoot regeneration in callus cultures derived from barley (*Hordeum vulgare* L.) immature embryos. Breed. Sci. 58, 129–135. doi: 10.1270/jsbbs.58.129

[B131] Rosspopoff O. Chelysheva L. Saffar J. Lecorgne L. Gey D. Caillieux E.. (2017). Direct conversion of root primordium into shoot meristem relies on timing of stem cell niche development. Development 144, 1187–1200. doi: 10.1242/dev.142570 28174250

[B132] Rukh G. Ahmad N. Rab A. Ahmad N. Fazal H. Akbar F.. (2019). Photo-dependent somatic embryogenesis from non-embryogenic calli and its polyphenolics content in high-valued medicinal plant of *Ajuga bracteosa* . J. Photochem. Photobiol. B. 190, 59–65. doi: 10.1016/j.jphotobiol.2018.11.012 30500677

[B133] Sae-Tang W. Heuvelink E. Kohlen W. Argyri E. Nicole C. Marcelis L. (2024). Effect of far-red and blue light on rooting in medicinal cannabis cuttings and related changes in endogenous auxin and carbohydrates. Sci. Hortic. 325, 112624. doi: 10.1016/j.scienta.2023.112614

[B134] Saitou T. Tokutomi S. Harada H. Kamada H. (1999). Overexpression of phytochrome A enhances the light-induced formation of adventitious shoots on horse-radish hairy roots. Plant Cell Rep. 18, 754–758. doi: 10.1007/s002990050655

[B135] Sang Y. L. Cheng Z. J. Zhang X. S. (2018). iPSCs: A comparison between animals and plants. Trends Plant Sci. 23, 660–666. doi: 10.1016/j.tplants.2018.05.008 29880405

[B136] Sena G. Wang X. Liu H.-Y. Hofhuis H. Birnbaum K. D. (2009). Organ regeneration does not require a functional stem cell niche in plants. Nature 457, 1150–1153. doi: 10.1038/nature07597 19182776 PMC2649681

[B137] Shah K. An N. Kamanova S. Chen L. Jia P. Zhang C.. (2021). Regulation of flowering time by improving leaf health markers and expansion by salicylic acid treatment: A new approach to induce flowering in Malus domestica. Front. Plant Sci. 12. doi: 10.3389/fpls.2021.655974 PMC8328039 34349772

[B138] Shah K. Zhu X. Zhang T. Chen J. Chen J. Qin Y. (2024). The poetry of nitrogen and carbon metabolic shifts: The role of C/N in pitaya phase change. Plant Sci. 348, 112240. doi: 10.1016/j.plantsci.2024.112240 39208994

[B139] Shen Y. Fan K. Wang Y. Wang H. Ding S. Song D.. (2022). Red and blue light affect the formation of adventitious roots of tea cuttings (Camellia sinensis) by regulating hormone synthesis and signal transduction pathways of mature leaves. Front. Plant Sci. 13. doi: 10.3389/fpls.2022.943662 PMC9301306 35873958

[B140] Shi X. Dai X. Liu G. Bao M. (2009). Enhancement of somatic embryogenesis in camphor tree (*Cinnamomum camphora* L.): osmotic stress and other factors affecting somatic embryo formation on hormone-free medium. Trees 23, 1033–1042. doi: 10.1007/s00468-009-0345-9

[B141] Shim S. Lee H. G. Seo P. J. (2021). MET1-dependent DNA methylation represses light signaling and influences plant regeneration in *Arabidopsis* . Mol. Cells 44, 746–757. doi: 10.14348/molcells.2021.0160 34711691 PMC8560584

[B142] Siddique A. B. Islam S. S. (2018). Effect of light and dark on callus induction and regeneration in tobacco (*Nicotiana tabacum* L.). Bangl. J. Bot. 44, 643–651. doi: 10.3329/bjb.v44i4.38636

[B143] Siegień I. Adamczuk A. Wróblewska K. (2013). Light affects *in vitro* organogenesis of *Linum usitatissimum* L. and its cyanogenic potential. Acta Physiol. Plant 35, 781–789. doi: 10.1007/s11738-012-1118-4 25834293 PMC4372823

[B144] Sivanesan I. Lim M. Y. Jeong B. R. (2011). Somatic embryogenesis and plant regeneration from leaf and petiole explants of *Campanula punctata* Lam. var. *rubriflora* Makino. Plant Cell Tiss Organ Cult. 107, 365–369. doi: 10.1007/s11240-011-9983-x

[B145] Skoog F. Miller C. (1957). Chemical regulation of growth and organ formation in plant tissues cultured in vitro. Symp Soc Exp Biol. 11, 118–30. 13486467

[B146] Song X. Guo P. Xia K. Wang M. Liu Y. Chen L.. (2023). Spatial transcriptomics reveals light-induced chlorenchyma cells involved in promoting shoot regeneration in tomato callus. Proc. Natl. Acad. Sci. U.S.A. 120, e2310163120. doi: 10.1073/pnas.2310163120 PMC10515167 37703282

[B147] Sorin C. Bussell J. D. Camus I. Ljung K. Kowalczyk M. Geiss G.. (2005). Auxin and light control of adventitious rooting in *Arabidopsis* require ARGONAUTE1. Plant Cell. 17, 1343–1359. doi: 10.1105/tpc.105.031625 15829601 PMC1091759

[B148] Sorin C. Negroni L. Balliau T. Corti H. Jacquemot M.-P. Davanture M.. (2006). Proteomic analysis of different mutant genotypes of *Arabidopsis* led to the identification of 11 proteins correlating with adventitious root development. Plant Physiol. 140, 349–364. doi: 10.1104/pp.105.067868 16377752 PMC1326056

[B149] Steward F. C. Mapes I. I. M. O. Mears K. (1958). Growth And organized development of cultured cells. II. organization in cultures grown from freely suspended cell. Am. J. Bot. 45, 705–708. doi: 10.1002/j.1537-2197.1958.tb10599.x

[B150] Stone S. L. Braybrook S. A. Paula S. L. Kwong L. W. Meuser J. Pelletier J.. (2008). *Arabidopsis* LEAFY COTYLEDON2 induces maturation traits and auxin activity: Implications for somatic embryogenesis. Proc. Natl. Acad. Sci. U.S.A. 105, 3151–3156. doi: 10.1073/pnas.0712364105 18287041 PMC2268600

[B151] Sugimoto K. Jiao Y. Meyerowitz E. M. (2010). *Arabidopsis* regeneration from multiple tissues occurs via a root development pathway. Dev. Cell. 18, 463–471. doi: 10.1016/j.devcel.2010.02.004 20230752

[B152] Sui L. Peng M. Kong L. Que W. Yuan H. Zhang Y. (2021). Effects of light on callus multiplication of *Actinidia Arguta* . IOP Conf. Ser.: Earth Environ. Sci. 784, 12027. doi: 10.1088/1755-1315/784/1/012027

[B153] Sukumar P. (2010). The role of auxin and ethylene in adventitious root formation in Arabidopsis and tomato. PHD thesis.

[B154] Takahashi F. Sato-Nara K. Kobayashi K. Suzuki M. Suzuki H. (2003). Sugar-induced adventitious roots in *Arabidopsis* seedlings. J. Plant Res. 116, 83–91. doi: 10.1007/s10265-002-0074-2 12736780

[B155] Temman H. Sakamoto T. Ueda M. Sugimoto K. Migihashi M. Yamamoto K.. (2023). Histone deacetylation regulates *de novo* shoot regeneration. Proc. Natl. Acad. Sci. U.S.A. 2, pgad002. doi: 10.1093/pnasnexus/pgad002 PMC9944245 36845349

[B156] Tiidema A. Truve E. (2004). Efficient regeneration of fertile barley plants from callus cultures of several Nordic cultivars. Hereditas. 140 (3), 171–6. doi: 10.1111/j.1601-5223.2004.01757.x 15198706

[B157] Tombesi S. Palliotti A. Poni S. Farinelli D. (2015). Influence of light and shoot development stage on leaf photosynthesis and carbohydrate status during the adventitious root formation in cuttings of *Corylus avellana* L. Front. Plant Sci. 6. doi: 10.3389/fpls.2015.00973 PMC4654426 26635821

[B158] Valencia-Lozano E. Cabrera-Ponce J. L. Barraza A. López-Calleja A. C. García-Vázquez E. Rivera-Toro D. M. (2024). Editing of SlWRKY29 by CRISPR-activation promotes somatic embryogenesis in Solanum lycopersicum cv. Micro-Tom. PLoS One. 19 (4), e0301169. doi: 10.1371/journal.pone.0301169 38557903 PMC10984418

[B159] Van The Vinh B. Tung H. T. Bien L. T. Khai H. D. Mai N. T. N. Luan V. Q.. (2023). Stem elongation and somatic embryogenesis under red light-emitting diode and subsequent growth of tuberous begonias (*Begonia × tuberhybrida* Voss) plantlets on medium containing cobalt nano-particles. Plant Cell Tiss Organ Cult. 155, 553–566. doi: 10.1007/s11240-023-02519-1

[B160] Verstraeten I. Beeckman T. Geelen D. (2013). Adventitious root induction in *Arabidopsis thaliana* as a model for *in vitro* root organogenesis. Methods Mol. Biol. 959, 159–175. doi: 10.1007/978-1-62703-221-6_10 23299674

[B161] Verstraeten I. Buyle H. Werbrouck S. Van Labeke M. C. Geelen D. (2020). *In vitro* shoot growth and adventitious rooting of *Wikstroemia gemmata* depends on light quality. Israel J. Plant Sci. 67, 16–26. doi: 10.1163/22238980-20191110

[B162] Wang H. Caruso L. V. Downie A. B. Perry S. E. (2004). The embryo MADS domain protein AGAMOUS-Like 15 directly regulates expression of a gene encoding an enzyme involved in gibberellin metabolism. Plant Cell. 16, 1206–1219. doi: 10.1105/tpc.021261 15084721 PMC423210

[B163] Wang W. Liu J. Wang H. Li T. Zhao H. (2021). A highly efficient regeneration, genetic transformation system and induction of targeted mutations using CRISPR/Cas9 in *Lycium ruthenicum* . Plant Methods 17, 71. doi: 10.1186/s13007-021-00774-x 34217355 PMC8254353

[B164] Wang H. Liu H. Wang W. Zu Y. (2008). Effects of Thidiazuron, basal medium and light quality on adventitious shoot regeneration from *in vitro* cultured stem of *Populus alba×P. berolinensis* . J. Forestry Res. 19, 257–259. doi: 10.1007/s11676-008-0042-3

[B165] Wang F.-X. Shang G.-D. Wu L.-Y. Xu Z.-G. Zhao X.-Y. Wang J.-W. (2020). Chromatin accessibility dynamics and a hierarchical transcriptional regulatory network structure for plant somatic embryogenesis. Dev. Cell 54, 742–757.e8. doi: 10.1016/j.devcel.2020.07.003 32755547

[B166] Wei X. Ding Y. Wang Y. Li F. Ge X. (2020). Early low-fluence red light or darkness modulates the shoot regeneration capacity of excised *Arabidopsis* roots. Plants 9, 1378. doi: 10.3390/plants9101378 33081176 PMC7602781

[B167] Weijers D. Wagner D. (2016). Transcriptional responses to the auxin hormone. Annu. Rev. Plant Biol. 67, 539–574. doi: 10.1146/annurev-arplant-043015-112122 26905654

[B168] Wójcikowska B. Botor M. Morończyk J. Wójcik A. M. Nodzyński T. Karcz J.. (2018). Trichostatin A triggers an embryogenic transition in *Arabidopsis* explants via an auxin-related pathway. Front. Plant Sci. 9. doi: 10.3389/fpls.2018.01353 PMC6146766 30271420

[B169] Wu X. Zhang X. Wang Y. Wu C. Sun Y. Zhang Y.. (2023). Additional far-red light promotes adventitious rooting of double-root-cutting grafted watermelon seedlings. Hortic. Plant J. 10, 1424–1436. doi: 10.1016/j.hpj.2022.11.012

[B170] Wynne J. McDonald M. S. (2002). Adventitious root formation in woody plant tissue: The influence of light and indole-3-butyric acid (IBA) on adventitious root induction in *Betula Pendula* . In Vitro Cell. Dev. Biol. – Plant 38, 210–212. doi: 10.1079/IVPIVP2001266

[B171] Xing Z. Y. Yuan H. Y. Wang L. F. Zheng L. P. (2008). Regenerating plants from *in vitro* culture of *Erigeron breviscapus* leaves. Plant Cell Rep. 27, 39–45. doi: 10.1007/978-3-031-13388-6_8 17938931

[B172] Xu L. (2018). *De novo* root regeneration from leaf explants: wounding, auxin, and cell fate transition. Curr. Opin. Plant Biol. 41, 39–45. doi: 10.1016/j.pbi.2017.08.004 28865805

[B173] Xu D. (2020). COP1 and BBXs-HY5-mediated light signal transduction in plants. New Phytol. 228, 1748–1753. doi: 10.1111/nph.16296 31664720

[B174] Xu C. Cao H. Xu E. Zhang S. Hu Y. (2018a). Genome-wide identification of *Arabidopsis* LBD29 target genes reveals the molecular events behind auxin-induced cell reprogramming during callus formation. Plant Cell Physiol. 59, 749–760. doi: 10.1093/pcp/pcx168 29121271

[B175] Xu C. Cao H. Zhang Q. Wang H. Xin W. Xu E.. (2018b). Control of auxin-induced callus formation by bZIP59–LBD complex in *Arabidopsis* regeneration. Nat. Plants. 4, 108–115. doi: 10.1038/s41477-017-0095-4 29358751

[B176] Xu C. Chang P. Guo S. Yang X. Liu X. Sui B.. (2023). Transcriptional activation by WRKY23 and derepression by removal of bHLH041 coordinately establish callus pluripotency in *Arabidopsis* regeneration. Plant Cell. 36, 158–173. doi: 10.1093/plcell/koad255 37804093 PMC10734573

[B177] Xu Y. Yang M. Cheng F. Liu S. Liang Y. (2020). Effects of LED photoperiods and light qualities on *in vitro* growth and chlorophyll fluorescence of *Cunninghamia lanceolata* . BMC Plant Biol. 20, 269. doi: 10.1186/s12870-020-02480-7 32517650 PMC7285490

[B178] Yamamoto N. Kobayashi H. Togashi T. Mori Y. Kikuchi K. Kuriyama K.. (2005). Formation of embryogenic cell clumps from carrot epidermal cells is suppressed by 5-azacytidine, a DNA methylation inhibitor. J. Plant Physiol. 162, 47–54. doi: 10.1016/j.jplph.2004.05.013 15700420

[B179] Yan Y. Luo H. Qin Y. Yan T. Jia J. Hou Y.. (2024). Light controls meso-phyll-specific post-transcriptional splicing of photo regulatory genes by AtPRMT5. Proc. Natl. Acad. Sci. U.S.A. 121, e2317408121. doi: 10.1073/pnas.2317408121 38285953 PMC10861865

[B180] Yang X. Zhang X. (2010). Regulation of somatic embryogenesis in higher plants. Crit. Rev. Plant Sci. 29, 36–57. doi: 10.1080/07352680903436291

[B181] Yu Y. Qin W. Li Y. Zhang C. Wang Y. Yang Z.. (2019). Red light promotes cot-ton embryogenic callus formation by influencing endogenous hormones, polyamines and antioxidative enzyme activities. Plant Growth Regul. 87, 187–199. doi: 10.1007/s10725-018-0461-x

[B182] Yuan Y. Bai M. Ni P. Li Y. Chang X. He J.. (2024). Comparative transcriptome profiling reveals that light coordinates auxin to inhibit adventitious root formation in grapevine. Hortic. Plant J., S2468014124000736. doi: 10.1016/j.hpj.2024.02.003

[B183] Yun F. Liu H. Deng Y. Hou X. Liao W. (2023). The role of light-regulated auxin signaling in root development. Int. J. Mol. Sci. 24, 5253. doi: 10.3390/ijms24065253 36982350 PMC10049345

[B184] Zdravković-Korać S. Belić M. Ćalić D. Milojević J. (2023). Somatic embryogenesis in spinach—A review. Acta Hortic. 9, 1048. doi: 10.3390/horticulturae9091048

[B185] Zeng Y. Schotte S. Trinh H. K. Verstraeten I. Li J. Van De Velde E.. (2022). Genetic dissection of light-regulated adventitious root induction in *Arabidopsis thaliana* hypocotyls. Int. J. Mol. Sci. 23, 5301. doi: 10.3390/ijms23105301 35628112 PMC9140560

[B186] Zeng L. Xu H. Zeng Y. Luan A. Wang H. (2009). High efficiency *in vitro* plant regeneration from epicotyl explants of *Ponkan Mandarin* (*Citrus reticulata* Blanco). In Vitro Cell. Dev. Biol.-Plant. 45, 559–564. doi: 10.1007/s11627-009-9248-0

[B187] Zhai S. Cai W. Xiang Z.-X. Chen C.-Y. Lu Y.-T. Yuan T.-T. (2021). PIN3-mediated auxin transport contributes to blue light-induced adventitious root formation in *Arabidopsis* . Plant Sci. 312, 111044. doi: 10.1016/j.plantsci.2021.111044 34620442

[B188] Zhai N. Xu L. (2021). Pluripotency acquisition in the middle cell layer of callus is required for organ regeneration. Nat. Plants. 7, 1453–1460. doi: 10.1038/s41477-021-01015-8 34782770

[B189] Zhang T. Ge Y. Cai G. Pan X. Xu L. (2023). WOX-ARF modules initiate different types of roots. Cell Rep. 42, 112966. doi: 10.1016/j.celrep.2023.112966 37556327

[B190] Zhang T.-Q. Lian H. Zhou C.-M. Xu L. Jiao Y. Wang J.-W. (2017). A two-step model for *de novo* activation of WUSCHEL during plant shoot regeneration. Plant Cell. 29, 1073–1087. doi: 10.1105/tpc.16.00863 28389585 PMC5466026

[B191] Zhang C. Tang Y. Tang S. Chen L. Li T. Yuan H.. (2024). An inducible CRISPR activation tool for accelerating plant regeneration. Plant Commun. 5, 100823. doi: 10.1016/j.xplc.2024.100823 38243597 PMC11121170

[B192] Zhang G. Zhao F. Chen L. Pan Y. Sun L. Bao N.. (2019). Jasmonate-mediated wound signalling promotes plant regeneration. Nat. Plants. 5, 491–497. doi: 10.1038/s41477-019-0408-x 31011153

[B193] Zheng Y. Ren N. Wang H. Stromberg A. J. Perry S. E. (2009). Global identification of targets of the *Arabidopsis* MADS domain protein AGAMOUS-Like15. Plant Cell. 21, 2563–2577. doi: 10.1105/tpc.109.068890 19767455 PMC2768919

[B194] Zheng Q. Zheng Y. Ji H. Burnie W. Perry S. E. (2016). Gene regulation by the AGL15 transcription factor reveals hormone interactions in somatic embryogenesis. Plant Physiol. 172, 2374–2387. doi: 10.1104/pp.16.00564 27794101 PMC5129705

[B195] Zhou X. Zheng R. Liu G. Xu Y. Zhou Y. Laux T.. (2017). Desiccation treatment and endogenous IAA levels are key factors influencing high frequency somatic embryogenesis in *Cunninghamia lanceolata* (Lamb.) Hook. Front. Plant Sci. 8. doi: 10.3389/fpls.2017.02054 PMC5723420 29259612

[B196] Zuo Z. Liu H. Liu B. Liu X. Lin C. (2011). Blue light-dependent interaction of CRY2 with SPA1 regulates COP1 activity and floral initiation in *Arabidopsis* . Curr. Biol. 21, 841–847. doi: 10.1016/j.cub.2011.03.048 21514160 PMC3150455

